# Proximate Composition, Health Benefits, and Food Applications in Bakery Products of Purple-Fleshed Sweet Potato (*Ipomoea batatas* L.) and Its By-Products: A Comprehensive Review

**DOI:** 10.3390/antiox13080954

**Published:** 2024-08-06

**Authors:** María de los Ángeles Rosell, Jhazmin Quizhpe, Pablo Ayuso, Rocío Peñalver, Gema Nieto

**Affiliations:** Department of Food Technology, Nutrition and Food Science, Veterinary Faculty, University of Murcia, Regional Campus of International Excellence “Campus Mare Nostrum”, Campus de Espinardo, 30100 Murcia, Spain; mariaangeles.rosellp@um.es (M.d.l.Á.R.); jhazminedith.quizhper@um.es (J.Q.); pablo.ayuson@um.es (P.A.); rocio.penalver@um.es (R.P.)

**Keywords:** sweet potato, *Ipomoea batatas* (L.) Lam., food application, antioxidants, anthocyanins

## Abstract

*Ipomoea batatas* (L.) Lam is a dicotyledonous plant originally from tropical regions, with China and Spain acting as the main producers from outside and within the EU, respectively. The root, including only flesh, is the edible part, and the peel, leaves, stems, or shoots are considered by-products, which are generated due to being discarded in the field and during processing. Therefore, this study aimed to perform a comprehensive review of the nutritional value, phytochemical composition, and health-promoting activities of purple-fleshed sweet potato and its by-products, which lead to its potential applications in bakery products for the development of functional foods. The methodology is applied to the selected topic and is used to conduct the search, review abstracts and full texts, and discuss the results using different general databases. The studies suggested that purple-fleshed sweet potato parts are characterized by a high content of essential minerals and bioactive compounds, including anthocyanins belonging to the cyanidin or the peonidin type. The flesh and leaves are also high in phenolic compounds and carotenoids such as lutein and β-carotene. The high content of phenolic compounds and anthocyanins provides the purple-fleshed sweet potato with high antioxidant and anti-inflammatory power due to the modulation effect of the transcription factor Nrf2 and NF-kB translocation, which may lead to protection against hepatic and neurological disorders, among others. Furthermore, purple-fleshed sweet potato and its by-products can play a dual role in food applications due to its attractive color and wide range of biological activities which enhance its nutritional profile. As a result, it is essential to harness the potential of the purple-fleshed sweet potato and its by-products that are generated during its processing through an appropriate agro-industrial valorization system.

## 1. Introduction

*Ipomoea batatas* (L.) Lam. or sweet potato (SP) is a dicotyledonous plant and herbaceous perennial vine that is native to the neotropics [1,2,3]. The migration of the plant spread its growth to 114 countries worldwide [4]. It belongs to the series *Ipomoea batatas*, which is taxonomically placed in the genus *Ipomoea*; recent research has found that 14 wild species are related to the SP. These are the following: *I. cordatotriloba* Dennstedt, *I. cynanchifolia* Meisn, *I. grandifolia* O’Donell, *I. lacunose* L., *I. leucantha* Jacquin, *I. littoralis* Blume, *I. ramosissima* Choisy, *I. splendor-sylvae* House, *I. tabascana* McDonald and Austin, *I. tenuissima* Choisy, *I. tiliacea* (Willd.) Choisy in D. C, *I. trifida* (H. B. K.) G. Don, and *I. triloba* L. [5]. All these species belong to the family *Convolvulaceae*, although *Ipomoea batatas* is the only known cropped and major-economic-importance species in the family [6].

**Figure 1:**
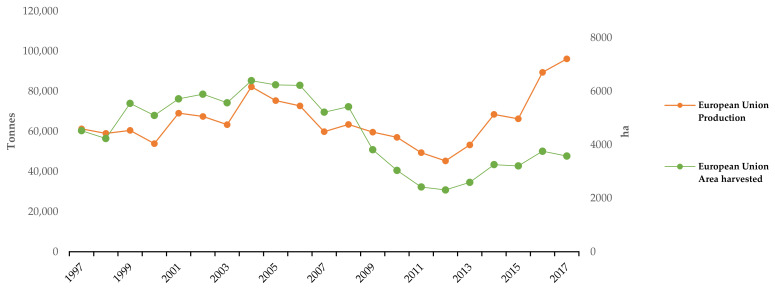
European Union production and harvested area of SP from 1997 to 2017 [8].

SP is the seventh most important crop worldwide [7]. According to FAOSTAT, the world production of SP was 86.4 million tons in 2022. The main destination of the total amount produced was the food supply (58%), followed by feed (32%) and waste (7%). Asia and Africa are the biggest SP producers, making up 61% and 34% of the world’s total production, respectively. The Chinese SP total production accounts for 54% of the total global production, while only 2% of the production belongs to industrialized countries, mainly in the USA and Japan [8]. In the European Union, SP production has increased since 2012, predicting an upward trend in future years (Figure 1). In this context, the latest data from the FAO database show Spain’s leadership as the largest SP producer in the EU, with 83 thousand tons being produced in 2022 [8]. In Spain, the crop is located, mainly, in Andalucía and Valencia, but also in Extremadura, Baleares, Aragón, and Murcia, raising its production to 79 thousand tons in 2021 [9]. Due to its neotropical origin, SP has been a traditional Mediterranean harvest crop. SP crops are grown as annual plants by vegetative propagation with a short growing period of 90 to 120 days [10]. The planting period requires a moderate temperature (21–26 °C), plenty of sunshine, sandy loam with clay subsoil, and a soil pH range between 5.5 and 6.5 [11]. However, SP is sensitive to salinity and alkalinity conditions [12,13].

**Figure 2:**
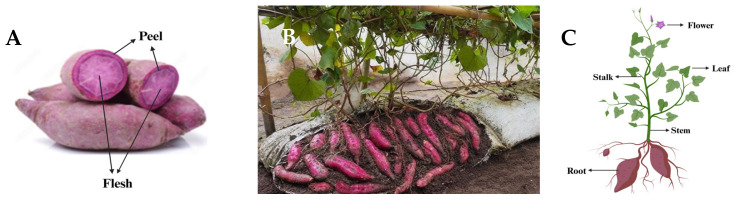
(**A**) Cross-section of a mature PFSP roots; (**B**) Cultivar of PFSP; (**C**) Different parts of a PFSP plant.

The SP cultivar has an elongated and tapered appearance, and this plant may produce 40–50 roots reaching an average length of 30 cm, with a weight between 100 and 1000 g, although it differs among commercial cultivars [14]. The edible part of the SP is the root, the most consumed worldwide and used as food or as a raw material for starch production [15]. However, SP leaves, young stems, and shoots are often discarded in the field or used as livestock feed [16]. In fact, these above-ground parts are also edible and consumed as a leafy or green vegetable in parts of Asia and Africa due to the richness in minerals, vitamins, proteins, pigments, and polyphenols [17,18]. The processing of SP generates a wide variety of bio-waste and by-products depending on whether they are a result of the agricultural phase or the industrial processing phase [19]. In the field, bio-waste is generated from the removal of leaves, young stems, and shoots and also from the root tubers that do not achieve size requirements or are damaged due to harvesting techniques [20]. During processing, by-products come from peels, trimming, chunks of tuber, and nutrient-rich wastewater (Figure 2) [21].

SP color has led to a divide among commercial cultivars, resulting in two categories, which depend on (i) the color of the skin and (ii) the flesh color. SP skin colors include white, cream, yellow, orange, pink, red, and purple. SP flesh colors include white, cream, yellow, orange, and purple. The differences between these commercial cultivars only extend to the bioactive and non-nutrient compounds [22]. Due to the wide variety of SP commercial cultivars, this review is going to focus on the study of purple-fleshed sweet potato (PFSP). The high accumulation of acylated anthocyanins in the root leads to the common purple color of the skin and the flesh [23,24].

The therapeutic and medicinal benefits of PFSP have been known since its domestication 5000 years ago, playing a significant role in combating food shortage and malnutrition because of its nutritive values and biological activities [25]. The high content of bioactive compounds, such as phenolic acids, anthocyanins, vitamins, dietary fiber, or resistant starch, provides purple-fleshed sweet potato with beneficial effects against a variety of diseases. Numerous studies have found that PFSPs present an anti-inflammatory [26], antioxidant [27], antimicrobial [28], antidiabetic [29], antimutation [30], anti-tumor [31], and hypouricemic [32] effect, as well as hepatic [33], and neuroprotective action [34]. On the other hand, new alternatives use for PFSP bio-wastes have been proposed to avoid environmental problems [35]. Purple-fleshed sweet potato by-products in combination with ultrasound and microwave extraction techniques can be a valuable raw material to obtain the pure bioactive compounds, anthocyanins and dietary fiber, for the manufacture of value-added products such as functional foods [36,37,38].

Due to the growing interest in research on agricultural by-products and its beneficial effects for applications in food industry, especially in bakery products, this article aims to review the existing literature about the functional characteristics, health benefits, and food application of purple-fleshed sweet potato.

## 2. Materials and Methods

This comprehensive review followed four steps: selecting the topic, conducting the literature search, reviewing abstracts and full texts, and discussing the results. For this purpose, the Science Direct, Google Scholar, PubMed, Web of Science, Scopus, and Dialnet databases were searched to recognize the appropriate studies, according to the review’s aim. The final search was conducted in July 2024 and included English and Spanish-language-based international articles, including reviews and research, reports, and theses. The keyword “purple-fleshed sweet potato” was utilized combined with other terms such as antioxidant, anti-inflammatory, bakery products, food waste, anthocyanins, phenolic acids, minerals, peel, or hepatoprotective effects. After the full search, duplicates were removed, and the abstracts and their specific sections of the articles were read to ensure that they addressed the review inclusion criteria. The eligible criteria were studies that analyzed PFSP in at least one of the three dimensions focused on in this review (nutritional characteristics, health benefits, and food application). Therefore, the studies of interest focusing on sweet potato, or sweet potato varieties different from the purple one, were summarized and synthesized to integrate into the comprehensive review. Finally, no specific platforms were necessary to document the comprehensive search due to the nature of this review.

## 3. Nutritional Characteristics

### 3.1. Proximate Composition of Purple-Fleshed Sweet Potato

There is a large variability in nutrients between the different botanical parts of PFSP (flesh, leaves, stems, stalks, shoots, or peels). Genetic [39], agricultural practices [40], geographic [41], maturation stage [42], and environmental factors also contribute to this variation. According to the U.S. Department of Agriculture [43], PFSP provides 85 kcal per 100 g of FW of the edible portion and is considered a high-calorie food due to its high moisture content (ranging from 62.6 to 73.6%) [44].

Carbohydrates represent up to 72.10 g/100 g (Table 1) of the dry weight (DW) of PFSP. Free sugars of low molecular weight or reducing sugars constitute a small fraction of the total carbohydrates, reporting ranges between 1.01 and 5.94 g/100 g depending on the variety or the maturity stage, with maltose, sucrose, and glucose + fructose being the predominant reducing sugars with 11.98, 8.33, and 6.52% of FW, respectively [45,46]. However, a large part of these constitute starches reaching values of 56.7 g/100 g of DW [47,48]. Native starch is composed of amylose and amylopectin, and its starch digestion rate depends on the proportion of amylose/amylopectin generating different absorption rates dividing starch into three categories: (i) rapidly digestible starch (RDS), (ii) slowly digestible starch (SDS), and (iii) resistant starch (RS) [49]. The results shown in Table 1 fit with the values reported by another study that compares the physicochemical characterization of seven PFSP varieties establishing an amylose content that varied from 18.2 to 27.2%, and RDS, SDS, and RS contents from 40.66% to 53.50%, from 10.40% to 23.84%, and from 29.25% to 43.50%, respectively [50]. Wang et al. [51] revealed that starch degradation provided abundant substrates for anthocyanin biosynthesis in PFSP roots since the most abundant PFSP starch, phosphorylase (SP), and phosphoglucomutase (PGM) promoted the synthesis of precursors for anthocyanin metabolism explaining the high anthocyanins content of PFSP (Section 3.2.1).

Dietary fiber is defined as a group of non-digestible carbohydrates that can lower blood glucose and act as substrates in the intestinal tract reducing digestive tract disorders, among other functions [52,53]. PFSP roots present around 16% DW total dietary fiber, having an insoluble fiber content higher than the soluble fiber content (Table 1). However, the content of this nutrient varies in other parts of the PFSP considered as by-products, being higher in peels containing more than 60% dietary fiber, of which 77.6% is insoluble dietary fiber [54].

Purple-fleshed sweet potato root has a low protein content of 2.33 g/100 g DW (Table 1), with the finding that the crude protein extracts are rich in amino acids indicating that glutamic acid, aspartate, arginine, alanine, and leucine had the five highest amino acids contents, with 565.75 mg/kg, 479.74 mg/kg, 413.54 mg/kg, 371.87 mg/kg, and 336.67 mg/kg, respectively [55,56]. Despite the low protein content of the flesh, some studies have revealed a high protein content reached in the leaves ranging from 16.2 to 30.3 g/100 g DW across diverse cultivars [57,58]. In addition, some studies have indicated that polysaccharides from PFSP contain proportions of proteins and uric acids that could enhance their antioxidant activities [59].

**Table 1:** Proximal composition of the edible part of PFSP (flesh).

Nutrient		Value	Ref.
Moisture	(g/100 g FW)	63.51 ± 0.20	[60]
Ash	(g/100 g DW)	2.06 ± 0.01	[61]
Proteins	(g/100 g DW)	2.33 ± 0.04	[62]
Crude Fat	(g/100 g DW)	0.51 ± 0.04	[63]
Total Dietary Fiber	(g/100 g DW)	15.8 ± 0.50	[64]
Insoluble Dietary Fiber	(g/100 g DW)	8.4 ± 1.30	[64]
Soluble Dietary Fiber	(g/100 g DW)	7.4 ± 1.40	[64]
Carbohydrates	(g/100 g DW)	79.10 ± 0.03	[65]
Starch	(g/100 g DW)	56.7 ± 0.35	[63]
RDS	(%)	46.1 ± 0.20	[66]
SDS	(%)	10.4 ± 0.20	[66]
RS	(%)	43.5 ± 0.20	[66]
Reducing Sugar	(g/100 g DW)	3.09 ± 0.01	[63]

**DW**: dry weight; **FW**: fresh weight; **RDS**: rapidly digestible starch; **SDS**: slowly digestible starch; **RS**: resistant starch.

### 3.2. Bioactive Compounds

More than 100 different bioactive compounds have been identified from diverse PFSP parts, including flesh, skin, and leaves, such as flavonoids, non-flavonoids, carotenoids, or organic acids. However, a large variety of these bioactive compounds show values that vary sharply in different PFSP varieties from trace to elevated content due to factors such as the genotype, harvest, postharvest, or extraction procedures [67]. The following sections described the major bioactive compounds located in PFSP.

#### 3.2.1. Anthocyanins

Anthocyanins, responsible for the color of purple-fleshed sweet potato, represent one of the most important constituent groups of PFSP. These compounds are secondary metabolites and water-soluble pigments belonging to the phenolic group with fundamental functions in the plant [68]. However, PFSP anthocyanins are usually glycated and acylated, with 3-sophoroside-5-glucoside and mono- or di-acylation with phenolic acids such as caffeic, ferulic, vanillic, *p*-coumaric, or *p*-hydroxybenzoic acids being the common glycation and acylation forms, respectively [69]. Concretely, these anthocyanins belong to the cyanidin or the peonidin type acylated with caffeic, ferulic, and *p*-hydroxybenzoic acids [70]. The acylation form guarantees heat stability, improving their application in heat-treated products [71]. Therefore, the importance of these compounds resides mainly in their function as antioxidant agents, being considered one of the most potent antioxidants of PFSP leaves, flesh, and peel [72,73].

The most abundant anthocyanins in PFSP are cyanidin 3-sophoroside-5-glucoside, cyanidin 3-(6,6′-dicaffeoyl-sophoroside)-5-glucoside, cyanidin 3-(6,6′-caffeoylphydroxybenzoyl sophoroside)-5-glucoside, cyanidin 3-(6,6′-caffeoylferuloylsophoroside)-5-glucoside, peonidin 3-(6,6′-dicaffeoylsophoroside)-5-glueoside, and peonidin 3-(6,6′-caffeoylphydroxybenzoyl sophoroside)-5-glucoside, which have been identified in diverse flesh, leaves, and peels as the richest anthocyanins. Although these cyanidins and peonidin derivatives are found mainly in purple sweet potato flesh [74], higher levels of cyanidin 3-(6″-feruloyl sophoroside)-5-glucoside and caffeoylated (cyanidin 3-sophoroside-5-glucoside) have been reported in leaves and peel [75]. Furthermore, the anthocyanins distribution in leaves and peel is similar to that in flesh with the former mainly consisting of cyanidin derivatives regardless of the cultivar.

However, relevant differences are appreciated in the supporting data indicating that TA seems higher in leaves and peels. In contrast, Su et al. [76] reported that the total contents of anthocyanins in PFSP *var* P40 leaves were much lower than those in the roots, suggesting an exceedingly diverse phenotype of anthocyanin biosynthesis between leaves and roots. This fact was attributed some years ago by Mano et al. [77] to the presence of a one-member transcriptional factor, *MYB*, which induces all structural anthocyanin biosynthesis genes, and is predominantly expressed in the roots but not in stems, leaves, or flowers either in the roots of orange-, yellow-, or white-fleshed varieties.

Finally, the increasing attention to PFSP anthocyanins, due to their high contents and multiple biological activities, has led to new anthocyanins constantly being identified in flesh, leaves, and peels, yet absent from stems [78,79].

#### 3.2.2. Phenolic Acids

A rich diversity of phenolic acids has been successfully quantified and identified in PFSP (Table 2). Around 30 phenolic acids and their derivatives have been reported, specifically hydroxybenzoic, chlorogenic, caffeic, ferulic, and *p*-coumaric acids are the primary phenolic acids in the flesh, leaves, and peels of purple sweet potato. Among the multiple functions they contribute, it has been shown how some phenolic compounds, such as ferulic and caffeic acids, increase anthocyanin stability via intermolecular co-pigmentation shielding the flavylium cation from nucleophilic attack by water and improving its functional structure [80,81].

Chlorogenic and caffeic acid and their derivatives are the most widely distributed phenolic acids in the leaves and peels of purple sweet potato, while salicylic and ferulic acids are more frequently present in the flesh. As mentioned, Jang et al. [98] quantified that 3,5-dicaffeoylquinic acid was the most abundant in the leaves of all varieties analyzed. Another research study conducted by Ooi et al. [99] evaluated the phenolic content of the skin and flesh from a purple sweet potato variety obtaining a similar total phenolic content in flesh and skin with 52.80 ± 0.84 and 48.19 ± 1.29 mg GAE/g, respectively. In addition, purple sweet potato varieties contain a significantly higher total phenolic content as compared to yellow and orange varieties due to genotype variations that influence the accumulation and types of synthesized phenolic acids [100,101].

Finally, phenolic acids are not distributed uniformly through the plant, suggesting that their distribution and content depend on a set of factors such as the extraction method, solvent, genotype, plant part, and environment, with flesh color being a relevant factor affecting the total content of phenols in sweet potatoes that could explain the wide range of data supported [102,103,104].

#### 3.2.3. Flavonols, Flavones, Carotenoids, and Other Bioactive Compounds

Non-anthocyanins flavonoids have also been identified in PFSP including flavonols, flavanes, and flavones [105]. A good source of flavonols and flavones is found in the flesh of the tuber where kaempferol, luteolin, and myricetin are the major constituents, while quercetin is mainly found in their leaves and peel. These compounds play significant biological regulatory functions such as their remarkable effect on protein regulation by reversibly combining with various proteins and enzymes in the body [106]. Furthermore, 18 organic acids from the roots of PFSP have been reported such as acetic, lactic, or pyruvic acids, although more studies are needed to quantify [107].

On the other hand, the considerable number of carotenoids located in the flesh and leaves of the tuber make it a valuable source of these compounds. Lutein, zeaxanthin, and carotene are the main carotenoid compounds identified. However, total carotenoid content varies depending on the extraction and drying method, as well as environmental factors where climate temperature influences the total carotenoid content in vegetables and fruit [108].

### 3.3. Minerals and Vitamins

PFSP and its by-products represent a rich source of essential minerals for the organism [58]. Compared to the mineral composition of other vegetables reported in the literature, purple-fleshed sweet potato presents a good source of Na, and mainly K, and Mg (Table 2) [109]. Deficiency of these minerals can lead to several metabolic disorders, such as DNA and RNA synthesis [110], neurological [111], and cardiovascular alterations [112]. The concentration of macro- and microelements varies depending on the botanical part of *I. batatas* (L.) Lam [113], although generally PFSP can cover part of the mineral requirements [86]. However, relevant differences could be attributed to genotype variation [41]. A significant mineral amount can be found in their flesh, leaves, and peel, with major values in most of the evaluated minerals in the peels compared to the flesh and leaves.

The purple-fleshed sweet potato has a high content of vitamins C and E (Table 2); although the higher contents are found in the flesh, some studies have found low quantities in peels and leaves. Furthermore, B group vitamins, such as B_1_, B_2_, and B_3_, have been detected in the flesh of the tuber in minor proportions. In addition, it has been shown that the content of vitamin C in PFSP is reduced after cooking methods such as boiling and frying with a loss of 72, and 61%, respectively [114]. Purple sweet potato has a high vitamin C content, reaching similar values to those of lemon, oranges, and grapefruits [115]. Vitamin C is involved in the biosynthesis of collagen, cholesterol metabolism, modulation of the iron pathway, and scavenging reactive oxygen and nitrogen species as part of its antioxidant mechanism [116].

## 4. Health Benefits

### 4.1. Antioxidant Activity

As described above, PFSP contains many bioactive compounds, such as caffeic, chlorogenic, and ferulic acid derivatives, naringenin, quercetin, and diverse anthocyanins. Apart from performing essential functions in the plant, these compounds, once ingested, can perform protective functions against the generation of ROS and oxidative damage, as well as playing a crucial role in the color stabilization of anthocyanins [117,118]. Even though ROS act as signaling molecules in physiological functions, the overproduction of ROS can damage lipids, membranes, proteins, or DNA, resulting in oxidative stress and damage [119,120]. Phenolic compounds provide purple-fleshed sweet potato with a high antioxidant capacity, showing significantly higher ABTS (2,2-azino-bis-3-(ethylbenzothiazoline-6-sulfonic acid)) and ORAC (oxygen radical absorbance capacity) activity, and up to 10 times higher DPPH (2,2-diphenyl-1-picrylhydrazyl) values than other sweet potato varieties such as white-fleshed, yellow-fleshed, or orange-fleshed sweet potato [121,122]. This high antioxidant capacity is mainly found in the peels, which contain the major levels of both total and individual phenolic compounds, although the rest of the PFSP by-products represent a good source of these phenolic compounds [75].

Several studies have observed the potential action of anthocyanins and polyphenols from PFSP against oxidative stress at in vitro levels in different types of cell lines. Esatbeyoglu et al. [123] reported that the application of three different polyphenol extracts from PFSP, mainly peonidin, and cyanidin derivatives, (1, 5, 10, 25, 50, and 75 µg/mL) in the Huh7 human cell line, resulting in a decrease in xanthine oxidase enzyme (XO), an enzyme involved in the generation of reactive oxygen species [124], and an increase in nuclear factor E2-related factor 2 transcription (Nrf2), a transcription factor that regulates the expression of genes involved in the oxidative stress response [125]. Ye et al. [126] observed similar results in the protective effects of PFSP anthocyanin on PC12 cells, obtaining a reduction in intracellular reactive oxygen species (ROS) generation and lipid peroxidation, in a dose-dependent manner. In addition, Insanu et al. [127] found that the higher content of phenolic acids and flavonoids had higher antioxidative activity, identifying that the highest antioxidative activity was in the leaves.

The antioxidant effect of PFSP has also been confirmed in in vivo experiments [128,129]. Zhang et al. [130] investigated the effects of HFD-induced rats treated with PFSP anthocyanin extracts daily for 6 weeks, reporting a level reduction in ROS and an inhibition of the receptor of advanced glycation end products (AGEs). It involved anti-obesity effects via attenuation of oxidative stress. Chang et al. [131] explored the effect of consumption of PFSP leaves on oxidative stress markers in healthy, nontrained, young male populations, revealing that consuming a high-polyphenol diet can modulate antioxidative status and decrease exercise-induced oxidative damage and pro-inflammatory cytokine secretion. In addition, Kano et al. [132] evaluated the antioxidative activity of anthocyanins from the PFSP cultivar *Ayamurasaki* in in vitro, and vivo trials, with rats and volunteers. The in vitro results suggested that the PFSP anthocyanin pigment showed higher radical-scavenging activity than ones from grape skin, elderberry, red cabbage, purple corn, and even ascorbic acid. Meanwhile, the in vivo results revealed that the urine of rats and humans that ingested PSFP increased their radical-scavenging activity.

However, a relevant point in in vivo experiments is the stability and antioxidant activity of these bioactive compounds after gastrointestinal digestion. Yang et al. [133] investigated the bioaccessibility and antioxidant activity of PFSP anthocyanins after intestinal digestion, obtaining a significant decline after digestion, although its stability depended on the type and number of acylated groups.

Several mechanisms explain the antioxidant action of the bioactive compounds present in PFSP. Oxidative stress is an imbalance between the production of ROS and their elimination by a protective mechanism [134]. Anthocyanins can induce the expression of antioxidants via the nuclear erythroid 2-related factor 2 (Nrf2) pathway and by reducing inflammation [135]. Polyphenols can reduce the catalytic activity of enzymes involved in ROS generation such as nitric oxide synthases (NOs), or XO [136,137]. However, the antioxidant capacity of these bioactive compounds can be attributed to their specific structural characteristics. Phenolic compounds and flavonoids react with ROS and thus terminate the chain reaction before cell viability is seriously affected [138]. For instance, chlorogenic acids possess one to two aromatic rings linked to hydroxyl groups, which lead to forming complexes with free radicals that are quickly broken down into further products that cannot generate any free radicals [139]. In fact, some studies have reported that antioxidant activity was positively correlated with the TPC of leaves and roots and the TA content of roots in sweet potato [140,141].

### 4.2. Hepatoprotective Action

The liver is an essential organ for a variety of physiological processes including macronutrients, alcohol, and drug metabolism, detoxification, endocrine control, cholesterol homeostasis, or immune defense [142]. Liver disease is the eleventh-leading cause of death annually and accounts for 4% of all deaths worldwide [143]. Dysfunction is associated with numerous liver diseases, such as alcohol-associated liver disease (AALD), non-alcoholic fatty liver disease (NAFLD), non-alcoholic steatohepatitis (NASH), drug-induced liver injury (DILI), cholestasis, viral hepatitis, and hepatocellular carcinoma (HCC) [144]. Recently, it has been suggested that the antioxidant and anti-inflammatory properties of PFSP bioactive constituents, such as phenolic compounds, anthocyanins, or polysaccharides, could prevent liver diseases by a reduction in alanine aminotransferase (ALT) and aspartate aminotransferase (AST) enzyme levels [145,146].

Polysaccharides exert hepatoprotective effects by modulating different signaling pathways. They downregulate key molecule expressions in the TLR4-P2X7R/NLRP3 signaling pathway, which controls inflammatory responses. Furthermore, polysaccharides activate the PI3K/AKT signaling pathway to recover redox balance and inhibit the expression of NF-kβ to mitigate pro-inflammatory cytokine expression (Table 3). This dual function ameliorates oxidative stress and liver inflammation [147]. The study by Sun et al. [148] observed changes in liver weight and size, increased scavenging activity and reducing power, and increased levels of superoxide dismutase (SOD), catalase (CAT), glutathione peroxidase (GSH-Px), and glutathione (GSH) after the consumption of PFSP polysaccharides for 31 days in mice, as well as a reduction in ALT and AST and the hepatic lipid peroxidation marker malondialdehyde (MDA). Another study observed the hepatoprotective effect of PFSP extracts in CCl_4_-induced oxidative hepatotoxicity fibrosis mice, reporting a reduction in serum levels of ALT, AST, and MDA, and increased SOD and GPx activity levels [149].

On the other hand, a relevant number of studies have found changes in the lipid profile after PFSP intake [150]. The reduction in triacylglycerides (TG) and total cholesterol (TC) decreases the lipid hepatic accumulation after the intake of PFSP leaves, suggesting that PFSP could exert a hypolipidemic action [151]. This effect could be attributed to the presence of chlorogenic acid, and its derivatives, a PFSP leaf constituent that regulates lipid metabolism by reducing preadipocyte differentiation and inhibiting fatty acids and total cholesterol synthesis [152,153,154]. Therefore, the hepatoprotective effect of PFSP extracts is achieved through multiples signaling pathways and mechanisms.

**Table 3:** Health benefits of different PFSP parts.

Type	Botanical Part	Experiment Design	Results	Ref.
Antioxidant activity				
Clinical trial	Leaves	Consumption of PSPL (200 g/day) by basketball players for 7 weeks	↑ Plasma polyphenol concentration, vitamin E and C levels, and LDL lag time	[129]
			↓ 8-OHdG	
Clinical trial	Leaves	Healthy adults were treated with PSPL (200 g/day) for 7 weeks	↑ LDL lag time, glutathione concentration, and urinary total phenol excretion	[128]
			↓ 8-OHdG	
In vivo	Flesh	Eight-week-old male Sprague Dawley strain rats were administered with 100, 200, and 400 mg of PFSP anthocyanin/kg b.w. once a day for 6 weeks	↓ ROS and AGESs	[130]
In vitro	Leaves	Evaluation of the inhibitory effect of PFSP leaves on endothelial cell-mediated LDL oxidation	↑ Free radical scavenging activity	[155]
			↑ Lag time for LDL oxidation	
In vitro	Stem, Leaves, and Flesh	Application of extracts from PFSP stems, leaves, and flesh to evaluate in vitro antioxidant activities by DPPH and CUPRAC assay	↑ DPPH and CUPRAC activity	[127]
			↑ TPC and TFC level	
In vitro	Flesh	Huh7 cells were treated with three different PFSP-derived polyphenol extracts (1, 5, 10, 25, 50, and 75 μg/mL) for 24 h	↓ α-amylase, α-glucosidase, and XO enzyme	[123]
			↑ Nrf2 factor and PON 1 transactivation	
In vitro	Flesh and Peel	Isolation and quantification of colorless caffeoyl compounds from PFSP to test their antioxidant abilities	↑ Total antioxidant capacity	[156]
			↑ Reducing power and DPPH	
In vivo	Flesh and Peel	Six healthy volunteers were administered with a PFSP beverage, rich in anthocyanins (2.49 mg/mL), collecting blood and urine samples at fixed times (0, 0.5, 1, 2, 3, 4, 6, 8, and 24 h) after feeding	↑ Urinary DPPH activity	[132]
In vitro	Flesh	PFSP species *Guijingshu 09-7* was treated to four different cooking methods (raw, boiling, roasting, and steaming)	↑ FRAP and DPPH	[157]
In vitro	Flesh	Utilization of PFSP anthocyanin (0, 0.1, 1, 10, 20, 40, 50, 80, 100, and 200 g/mL) on PC12 cells for 24 h	↓ Aβ-induced cytotoxicity and Ca^2+^ concentration	[158]
			↓ Intracellular ROS generation and LPO	
Clinical trial	Leaves	PSPL (200 g/day for lunch and dinner) was consumed by 15 healthy male volunteer for 5 weeks	↑ TPC and FRAP	[131]
			↓ TBARS, plasma PC, oxidative damage, and IL-6	
In vitro	Flesh	PFSP anthocyanins were isolated from *Ipomoea Batatas Poir Cv* (5–20 µg/mL) and administered in PC12 cells for 24 h	↓ Aβ-induced toxicity, ROS, and lipid peroxidation levels	[126]
			↓ Ca^2+^ intracellular concentration, and mitochondria dysfunction	
**Hepatoprotective action**				
In vivo	Leaves	Five-week-old male C57BL/6 mice received alcohol + PSPE (400 mg/kg bw for 7 days)	↓ ALT and AST enzyme levels	[151]
			↓ Blood alcohol concentration and inflammatory cells	
			↓ TG and TC levels	
In vivo	Flesh	Application of PSPP-1 (200 and 400 mg/kg; once daily until Day 28) in control mice (without any liver injury) and concanavalin A-induced liver injury mice	↓ ALT and AST enzyme levels	[147]
			↑ SOD and GSH levels	
			↓ MDA level	
			↓ TNF-α and IFN-γ levels	
In vivo	Flesh	Application of a novel polysaccharide (PSPP-A) extracted and isolated from PFSP in C57BL/6J male mice fed for 8 weeks with a high-fat diet blended with PSPP-A (100 mg/kg, 200 mg/kg and 400 mg/kg)	↓ Body weight and liver index	[145]
			↓ ALT and AST content	
			↓ TG and TC levels	
In vivo	Flesh	Female ICR mice were treated with three kinds of polysaccharides obtained from PFSP (100, 200 and 400 mg/kg bw of each extract per day) for 31 days	↓ Relative liver weight	[148]
			↓ ALT, AST, alkaline phosphatase and MDA levels	
			↑ SOD, CAT, and GSH-Px enzymes	
			↑ GSH and T-AOC levels	
In vivo	Flesh	Utilization of anthocyanins extract from PFSP (227.5, 455, and 910 mg/kg bw) in male mice after hepatic fibrosis induced by carbon tetrachloride for 3 weeks	↓ Relative liver weight	[149]
			↓ ALT, AST, and MDA levels	
			↑ SOD and GPx activity levels	
In vitro	Flesh	HepG2 hepatocytes were treated with AF (0, 50, 100, and 200 μg/mL)	↑ AMPK and ACC phosphorylation	[150]
			↓ TG and TC levels	
In vitro	Flesh	HepG2 cells were treated with raw, steamed, microwaving and roasted PFSP (100 μg/mL) for 24 h.	↓ ROS, GPx, and GR	[159]
			↑ GSH levels	
			↑ HO-1, NQO1, and GCLC expression	
**Anti-inflammatory effect**				
In vitro	Leaves	HAECs were treated with 100 μg/mL PSPLE for 24 h	↓ TNF-α-induced monocyte-endothelial cell adhesion	[160]
			↓ ERK1, and ERK2 expression	
			↓ VCAM-1, IL-8, and CD40 expression	
In vitro	Flesh	Effect of PFSP TNG 75 extracts ((1, 2, 3, 4, and 5 mg/mL) on RAW264.7 murine macrophage cells for 24 h	↓ NO production	[161]
			↓ NF-kβ, IL-6, and TNF-α levels	
In vitro	Flesh	Application of two anthocyanins, FAC-PSP and p-BAC-PSP (25, 50, 100, and 200 μg/mL), on RAW264.7 macrophages	↓ NO production level	[162]
			↓ NO production level	
In vivo	Flesh	(DSS)-induced colitis mice treated with 400 mg/kg of ASPP once per day for 30 days	↓ TNF-α release level	[163]
			↓ IL-1β, IL-6, and TNF-α	
In vitro	Leaves	Monosodium urate-induced RAW264.7 cells were treated with different concentrations (20, 40, 60 μg/mL) of PSPLP for 24 h	↑ SCFAs contents	[164]
			↓ IL-1β, IL-6, and TNF-α	
In vivo	Flesh	Male Wistar rats were given purple sweet potato extract (400 mg/kg/day for 9 days, once per day)	↓ IL-1β, MDA, COMP, and MMP-3 levels	[165]
			↑ chondrocytes	
In vitro	Leaves	Differentiated 3T3-L1 cells treated with PSPLE (0, 1, 2, and 4 mg/mL) for 72 h	↓ IL-6 and TNF-α expression	[166]
			↑ PARP and cellular apoptosis	
**Hypoglycemic and antidiabetic effect**				
In vivo	Leaves	Alloxan-induced diabetic male Wistar rats of 8–10 weeks old were treated with purple sweet potato leaves (50, 100, and 200 mg/kg bw) for 15 days	↓ MDA and blood glucose levels	[167]
			↑ Pancreatic histopathological features	
In vitro	Flesh	Utilization of three anthocyanins (3-caffeoyl-phydroxybenzoyl-sophoroside-5-glucoside, peonidin 3-caffeoyl sophoroside-5-glucoside, and peonidin 3-(6″-caffeoyl-6‴-feruloyl sophoroside)-5-glucoside) from PFSP (0, 10, and 50 μg/mL; 3 h) on human HepG2 cells	↓ Glucose production	[168]
In vivo	Flesh	Evaluating the effect of anthocyanin on fasting blood glucose levels in 6-week-old male C57BL/6 mice fed a 60% high-fat diet for 14 weeks	↓ Glucose production	[168]
In vivo	Flesh	Application of diacylated AF-PSPs (25 and 50 mg/kg bw) into free (SPF)-grade male Kun-Ming strain mice induced by a high-fructose/high-fat diet for nine weeks	↓ TG, TC, MDA, fasting blood glucose values, and blood glucose levels	[169]
			↑ T–SOD activity	
In vivo	Flesh	Male mice were fed with a high-fat diet and STZ to induce T2DM. The model mice were treated with 0, 227.5, 455, or 910 mg/kg bw of PSPA for ten days	↓ Blood glucose levels	[170]
			↑ GSH-Px level	
In vitro	Leaves	Application of four crude extracts (IBH, IBM, IBB, and IBW; 0.1 mg/mL) in 3T3-L1 preadipocytes	↑ PI3K, AKT, and Glut4 phosphorylation	[171]
			↑ Glucose uptake	
Clinical trial	Flesh and Peel	Double blinded pre-post test control group design in patients with T2DM (75 mL of PFSP that contained 11 g of anthocyanins—3 times per day 30 min after meal) for 4 weeks	↓ MDA	[172]
			↓ Fasting plasma glucose and 2hpppg levels	
			↓ Glycated albumin level	
**Neuroprotective effect**				
In vivo	Flesh	Application of PFSP anthocyanin (700 mg/kg/day) in eight-week-old C57BL 6J mice for 20 weeks by oral gavage	↑ Memory function, HFD-induced impairment mouse behavior, and IL-10 level	[173]
			↓ Body weight, fat content, hyperlipemia, and endotoxin level	
			↓ COX-2, TNF-α, IL-1β, IL-6, iNOS, ERK, JNK, and NF-kβ	
In vivo	Flesh	Utilization of purple sweet potato water extract (200 mg/kg bw*day) in d-galactose-induced male Wistar rats for 70 days	↓ TNF-α, p53, and GFAP expression	[174]
			↑ BDNF levels and spatial working memory	
In vitro	Leaves	BV-2 microglia cells were treated with purple sweet potato leaf extract (10–200 µg/mL) for 24 h	↓ NO, iNOS, COX-2, and TNF-α levels	[175]
In vivo	Flesh	15-month-old D-galactose-induced male Kunming mice were treated with PFSP anthocyanins (500 mg/kg*day bw) for 8 weeks by oral gavage	↓ Step-through latency, AGEs, Cu/Zn-SOD, and CAT activity	[176]
			↑ Spatial learning and memory ability	
			↓ JNK and cytochrome c levels	
In vivo	Flesh	Anthocyanins extracted from “Balinese” cultivar of PFSP administered to rat models of induced ischemic stroke	↑ Bcl-2 expression	[177]
			↓ Cytochrome C, caspase-3 levels, and apoptosis rate	
In vivo	Flesh	9-week-old male Kunming mice induced by D-galactose were administrated with PFSP anthocyanins (100 mg/kg*day) for 4 weeks via the oral route	↓ GFAP, COX-2, NF-kβ, and iNOS expression	[178]
			↓ MDA content	
			↑ Cu/Zn-SOD and CAT activity	
**Antimicrobial and prebiotic activity**				
In vitro	Flesh	Application of five peonidin-based anthocyanins from PFSP (0, 0.5, 1, 1.5, 2, and 2.5 mg/mL) to test the growth of probiotics and harmful bacteria	↑ *Bifidobacterium bifidum*, *Bifidobacterium adolescentis*, *Bifidobacterium infantis*, and *Lactobacillus acidophilus*	[179]
			↓ *Staphylococcus aureus* and *Salmonella typhimurium*	
In vivo	Flesh and peel	Three polysaccharides were extracted from PFSP and administered in female ICR mice (400 mg/kg bw) for 30 days by oral gavage	↑ *Bacteroidetes*, *Ruminococcaceae*, *Lachnospiraceae*, *Ruminococcus*, and *Oscillospir*	[180]
			↓ *Firmicutes*, *Proteobacteria*, *Alcaligenaceae*, and *Sutterella*	
In vitro	Flesh and Peel	Utilization of PFSP anthocyanin to evaluate the modulatory effect on human intestinal microbiota using fecal samples from volunteers (1% *w*/*v*)	↑ *Bifidobacterium* and *Lactobacillus/Enterococcus* spp.	[181]
			↓ *Bacteroides-Prevotella* and *Clostridium histolyticum*	
			↑ SCFA concentration	
In vivo	Flesh	7-week-old male Fischer 344 rats were treated with PFSP polyphenols (1% bw) for 27 days	↑ *Dorea*, cecal mucin, and cecal IgA level	[182]
			↓ *Oscillospira* and *Bacteroides*, and indole production	
In vitro	Flesh	Assessment of PFSP polyphenols (0.16%) by colonic fermentation using pig colonic digest under anaerobic conditions at 37 °C for 48 h	↑ *Eubacterium* spp., *Lactobacillus* spp., *Bifidobacterium* spp., *Collinsella stercoris*, and *Bulleidia* p1630cJ	[183]
			↓ *Clostridium* spp. and *Acidaminococcus* spp.	
**Hypouricemic action**				
In vivo	Flesh	SPF grade 8-week male Kun-Ming induced-hyperuricemia mice were treated with PFSP anthocyanins (25 mg/kg bw) and allopurinol (2.5 and 5 mg/kg bw)	↓ Serum uric acid level	[184]
			↓ TNF-α, IL-1β, IL-6, and TGF-β1 expression	
In vivo	Flesh	Oral application of PFSP anthocyanins (100 mg/kg bw) in three-week-old potassium oxonate-induced hyperuricemia ICR male mice	↓ Serum acid uric concentration	[185]
In vitro	Flesh and Peel	PFSP anthocyanins were evaluated for their inhibitory activity on commercial XO by spectrophotometrically measuring the formation of UA	↑ Inhibition XO activity rate	[186]
In vivo	Flesh and Peel	Hyperuricemia mice were administered with PFSP anthocyanins (25 and 100 mg/kg bw) orally for 7 days	↓ Uric acid level	[32]
			↓ 5′-NT and XO enzyme activity	
In vivo	Flesh	Utilization of an anthocyanin-rich purple sweet potato extract (75, 150, and 300 mg/kg bw, once daily) in potassium oxonate-induced hyperuricemia male Kun-Ming strain mice for 7 days	↓ Serum uric acid level	[187]
			↓ BUN and Cr levels	
**Antitumoral and antimutation activity**				
In vitro	Flesh	NALM6 human B-ALL cells were treated with PFSP anthocyanins (0, 20, 40, and 60 μg/mL) for 24 h	↓ NALM6 cell viability and S100A4 protein expression	[188]
			↑ NALM6 cells apoptosis and p38	
In vitro	Flesh	Utilization of three polysaccharides, PSPP1-1, PSPP2-1, and PSPP3-1, isolated from PFSP (100, 200, 300, 400, 500 μg/mL for SGC7901; 200, 400, 600, 800, 1000 μg/mL for SW620) on SGC7901 and SW620 tumor cells	↑ % Inhibition of tumor cells rate	[59]
In vitro	Leaves and Flesh	Application of anthocyanins isolated from the PFSP cultivar *Bhu Krishma* and the leaves of accession *S-1467* (100, 200, and 400 μg/mL) in human mammalian epithelial cells (MCF-10A)	↑ MCF-7, HeLa, and HCT-116 cells’ apoptosis	[189]
In vitro	Flesh	PFSP glucan was extracted and tested (0, 15.625, 31.25, 62.5, 125, 250, 500, and 1000 μg/mL) on HepG2, LOVO, MCF-7, LO2, GES-1, MCF-10A, NCM460, SGC-7901, and HGC-27 cells for 72 h	↑ % Inhibition in liver, colonic, and breast cells	[190]
In vitro	Flesh	Human colon cancer HT-29 cells were treated with PFSP polysaccharide (0, 10, 20, 40, 80, 160, and 320 μg/mL) for 24, 36, and 48 h	↓ Tumor cell viability	[191]
In vivo	Flesh	Evaluation of PFSP anthocyanin (100, 500, or 1000 mg/kg bw) in SPF-grade ICR mice implanted with mice S180 anal sarcoma cells for 5 weeks by oral gavage	↑ % Inhibition of tumor cells rate	[192]
In vivo	Flesh and Peel	C57BL/6J-APC^MIN/+^ mice were treated with purple sweet potato flesh and peel (10%) for 18 weeks	↓ Adenoma number	[193]

FAC-PSP: free anthocyanin compounds from purple sweet potato; p-BAC-PSP: protein-bound anthocyanin compounds from purple sweet potato; NO: nitric oxide; TNF-α: tumor necrosis factor-α; PSPP1-1: purple sweet potato polysaccharide composed of rhamnose, xylose, glucose, and galactose and their corresponding molar ratios of 17.54:1.00:2.67:1.10; PSPP2-1: purple sweet potato polysaccharide composed of rhamnose and galactose; PSPP3-1: purple sweet potato polysaccharide composed of rhamnose, xylose, glucose, and galactose, and their corresponding molar ratios of 3.51:1.92:1.44:1.00; DSS: dextran sulphate sodium; ASPP: alkali-soluble polysaccharide from purple flesh sweet potato; IL: interleukin; PSPLP: purple sweet potato leaf polyphenols; PSPP-1: purple sweet potato polysaccharide of glucose, galacturonic acid, galactose, arabinose, rhamnose, and glucuronic acid (molar ratio 320:20:19:10:8:2); ALT: alanine aminotransferase; AST: aspartate aminotransferase; IFN-γ: interferon-γ; T-SOD/SOD: total superoxide dismutase; GSH: glutathione; MDA: malondialdehyde; PSPP-A: purple sweet potato polysaccharide composed of L-rhamnose, D-arabinose, D-galactose, D-glucose, and D-glucuronic acid (molar ratios 1.89:8.45:1.95:1.13:1); TC: total cholesterol; TG: triglyceride; bw: body weight; ICR: Institute for Cancer Research; T-AOC: total antioxidant capacity; CAT: catalase; GSH-Px/GPx: glutathione peroxidase; AF: anthocyanin fraction from purple-fleshed sweet potato; AMPK: adenosine monophosphate-activated protein kinase; ACC: acetyl-coenzyme A carboxylase; ROS: reactive oxygen species; GR: glutathione reductase; HO-1: heme oxygenase-1; NQO_1_: NAD(P)H quinone oxidoreductase 1; GCLC: gamma glutamate-cysteine ligase; AF-PSPs: diacylated anthocyanins from purple sweet potato; AGESs: advanced glycation end products; COMP: cartilage oligomeric matrix protein; MMP-3: matrix metalloproteinase-3; LDL: low-density lipoprotein; CUPRAC: cupric reducing antioxidant capacity assay; TFC: total flavonoids content; IBH: n-hexane-fraction; IBM: 95% MeOH-fraction; IBB: n-BuOH-fraction; IBW: H_2_O-soluble fraction; PI3K: phosphoinositide 3 kinase; AKT: protein kinase B; Glut4: glucose transporter type 4; PSPLE: water-extracted purple sweet potato leaves authenticated by the National Plant Genetic Resources Centre of Taiwan Agricultural Research Institute with the account number Pin 375; PARP: cleaved caspase-3 and poly ADP-ribose polymerase; XO: xanthine oxidase enzyme; Nrf2: nuclear factor E2-related factor 2 transcription; PON 1: paraoxonase 1 enzyme; PFSP TNG 75: purple-fleshed sweet potato var. “Tainung 73”; NF-*κβ*: Nuclear factor kappa-light-chain-enhancer of activated B cells; HAECs: human aortic endothelial cells; PSPLE/PSPL: purple sweet potato leaf extract/purple sweet potato leaves; ERK/ERK1/ERK2; extracellular signal-regulated kinase; VCAM-1: vascular cell adhesion molecule 1; CD40: Cluster of differentiation 40; Bcl-2: B-cell lymphoma 2; GFAP: glial fibrillary acidic protein; iNOS: inducible nitric oxide synthase; COX-2: cyclooxygenase-2; Cu/Zn-SOD: copper/zinc superoxide dismutase; 2hpppg: 2 h post-prandial plasma glucose levels; 2DM: type 2 diabetes mellitus; Aβ: β-amyloid peptide; LPO: lipid peroxidation; HepG2: human hepatoma cell line; LOVO: human colonic carcinoma cell line; MCF-7: human breast carcinoma cell line; LO2: human normal hepatocyte GES-1: human normal stomach mucosa epithelial cell line; MCF-10A: human normal breast epithelial cell line; NCM460: human normal colon epithelial cell; SGC-7901 and HGC-27: human gastric carcinoma cell lines; TBARS: thiobarbituric acid-reactive substance; Plasma PC: protein carbonyl, a marker of protein oxidation; HFD: high-fat diet; JNK: c-Jun N-terminal kinase; p53: tumor protein P53; GFAP: glial fibrillary acidic protein; BDNF: brain-derived neurotrophic factor; TGF-β1: Transforming growth factor β; BUN: serum blood urea nitrogen; Cr: creatine; SCFA: short-chain fatty acid; IgA: immunoglobulin A; 8-OHdG: urinary 8-hydroxy-2-deoxyguanosine; 5′-NT: 5′-nucleotidase enzyme; STZ: streptozotocin; DPPH: 2,2-diphenyl-1-picrylhydrazyl, ↑ increase; ↓ decrease.

### 4.3. Anti-Inflammatory Effect

Numerous research studies have exhibited that oxidative stress is correlated with the development of inflammation [194]. The inflammatory process is often triggered by factors such as diet, alcohol, smoking, insufficient sleep, infections, antibiotics, or dysfunction [195]. During the inflammation process, inflammatory mediators, ROS, and cytokines play crucial roles [196]. Several studies conducted in vivo and in vitro have shown evidence about how certain bioactive compounds obtained from PFSP report anti-inflammatory properties [113,197].

Jiang et al. [162] measured the anti-inflammatory effect of two anthocyanins, FAC-PSP and p-BAC-PSP, obtained from the root of PFSP after an in vitro experiment evaluating the effect on inflammatory markers in LPS-induced RAW264.7 macrophages. Both anthocyanins showed significant anti-inflammatory activity by attenuating LPS production of NO, TNF-α, and ROS. Another study reported the potential benefits of a novel ASPP from PFSP on inflammation after an in vivo intervention in (DSS)-induced colitis mice. The alkali-soluble polysaccharide significantly inhibited the IL-1β, IL-6, and TNF-α cytokine levels in colitis tissue mice [163]. Sun et al. [164] investigated the effect of polyphenols from purple potato leaves on hyperuricemia, and their results showed that PSPLP significantly reduced the secretion of pro-inflammatory cytokines (IL-1β, IL-6, and TNF-α) in a dose-dependent manner [32].

Many of the beneficial properties of PFSP are attributed to the large number of bioactive compounds present in their flesh, peel, leaves, or stems. The mechanism involved in the anti-inflammatory effect appears to be similar for every bioactive compound. For instance, the anti-inflammatory property related to the higher anthocyanin content resulted in reduced protein expression of TNF-α, IL-1β, and IL-6 in colonic cells due to the prevention of NF-kB translocation reducing phosphorylation levels and the gene expression of pro-inflammatory cytokines and mediators [198,199,200]. However, the anti-inflammatory property related to the higher complex polysaccharides content is related to their ability to produce SCFAs modulating gut microbiota since human microbiota is not capable of digesting these polysaccharides, lowering the inflammation status [201,202]. While chlorogenic acid, the main polyphenol substance in the leaves of PFSP, has a proven capacity to inhibit the production of pro-inflammatory cytokines and chemokines because chlorogenic acid significantly attenuates the nuclear translocation, with the inhibition of the NF-kB signaling pathway being the mechanism responsible for the suppression of pro-inflammatory cytokines [203].

### 4.4. Hypoglycemic and Antidiabetic Effect

Diabetes mellitus (DM) is a heterogeneous metabolic disorder characterized by a chronic hyperglycemia status; a physiologically abnormal condition caused by disturbed insulin secretion, insulin effect, or both [204,205]. Approximately 95% of patients with DM present type 2 diabetes (T2DM) based on insulin resistance and β-cell dysfunction [206,207].

Many studies have evidenced the ability of PFSP to show beneficial effects due to its hypoglycemic action [208]. Solihah et al. [167] evaluated the effects of PFSP leaves in alloxan-induced diabetic rats, obtaining positive results with a reduction in blood glucose levels. The treatment with the leaf extract also had significantly lower MDA levels and better pancreatic histopathological features than untreated animals. A study in T2DM patients with the consumption of PFSP tubers treated with three daily oral doses of 75 mL for four weeks showed similar results, improving fasting and two hours post-prandial plasma glucose, as well as reducing the glycated albumin level [172].

The possible mechanisms responsible for the hypoglycemic effects of PFSP are multiple and unknown. However, it has been evidenced that polyphenols, anthocyanins, and protein-bound anthocyanins can increase AMP-activated protein kinase (AMPK) leading to an increased level of glucose transporter type 2 (GLUT2), glucokinase protein (GK), and insulin receptor α (INSR) [57]. Acetylated anthocyanins, mainly peonidin and cyanidin, revealed a low glucose production using an in vivo model with high-fructose/high-fat diet-induced mice for nine weeks, as well as reduced TG, TC, and MDA concentrations [169]. The cyanidin 3-caffeoyl-p-hydroxybenzoylsophoroside-5-glucoside showed glucose tolerance improvement and inhibition of hepatic gluconeogenesis in in vitro and in vivo experiments [168]. These facts are attributed to the capacity of anthocyanins to show affinity with insulin-regulated glucose transporter 4 (GLUT 4) and the competition with glucose in the small intestine of rats [209]. As a result, the hypoglycemic and antidiabetic effect of PFSP is determined by the diverse bioactive compounds with different target actions.

### 4.5. Neuroprotective Effect

Neurodegenerative diseases (NDs) are a heterogeneous group of complex diseases characterized by neuronal loss and progressive degeneration of different areas of the nervous system, making aging the most important risk factor in the development of NDs [210,211]. The elderly have increased in the last years, accounting for 15% of the global population, and this will double over the next two decades [212,213]. Alzheimer’s disease (AD), Parkinson’s disease (PD), and amyotrophic lateral sclerosis (ALS) are the three most prevalent age-related neurodegenerative conditions in the elderly [214]. Although the action mechanism of these diseases has not been elucidated, a common feature is that neuronal damage is caused by the abnormal aggregation and deposition of proteins which alter specific molecular mechanisms leading to cell toxicity and degeneration [215].

In recent years, research has focused on the role of some PFSP bioactive compounds in the reduction in neuroinflammation [174,176,178]. Neuroinflammation is associated with NDs, with microglia and astrocytes being key regulators of inflammatory responses in the central nervous system (CNS). In response to neuronal damage, inflammatory mediators secreted by pro-inflammatory microglia, such as IL-1β or TNF-α, may activate pro-inflammatory astrocytes and induce an inflammatory response [216]. A recent study tested the effects of *I. batatas* L. purple-fleshed variety on neuroinflammation-induced male mice with an HFD [173], observing that the anthocyanins present in the tuber can significantly reduce inflammatory markers, such as IL-1β and IL-6, COX-2, TNF-α, body weight, fat content, hyperlipemia, and endotoxin level, as well as causing an improvement in memory function. Kang et al. [175] evaluated the neuroprotective effect of PFSP leaves in LPS-induced BV-2 microglial cells, reporting scavenging DPPH radicals in a dose-dependent manner, and also observing a reduction in the release of NO and the concentration of inflammatory mediators such as iNOS, COX-2, and TNF-α. In this way, the anthocyanins mechanism in neurodegenerative diseases has been studied and explained as the combination of four effects: (i) their radical scavenging ability to eliminate ROS and nitrogen reactive species (NRS) and their promotion of antioxidant enzymes, (ii) the inhibition of inflammatory pathways in the CNS, (iii) their cytoprotective and anti-apoptotic effects on neurons; and (iv) the promotion of cholinergic neurotransmission [216,217,218,219].

### 4.6. Antimicrobial and Prebiotic Action

Prebiotics have recently been redefined as a substrate that is selectively utilized by a host microorganism conferring a health benefit [220]. Some previous research studies have highlighted that bioactive compounds from PFSP can exert their anti-inflammatory effect by regulating the intestinal microbiota composition [221]. Concretely, two mechanisms are involved in this effect: (i) the reduction in intestinal pathogens, such as *Clostridium* spp. or *Staphylococcus* spp., which produce components and metabolites that can initiate the inflammatory response with the corresponding release of pro-inflammatory cytokines, and (ii) the improvement in the healthy gut microbiota profile promoting the proliferation of short-chain fatty acids (SCFAs) involved in the suppression of inflammatory pathways and strengthening of the intestinal barrier [222,223,224,225].

According to the above-mentioned, an in vitro fermentation was carried out using fecal samples from healthy volunteers to test the modulatory effect of purple-fleshed sweet potato anthocyanins on human intestinal microbiota, showing a significant increase in the proliferation of *Bifidobacterium* and *Lactobacillus*/*Enterococcus* spp. and the SCFAs level, as well as an inhibition of the growth of *Clostridium histolyticum* [181]. Another study revealed similar results using a pig colonic digest where the polyphenolic content of PFSP was responsible for the drastic reduction in putrefactive products, especially *p*-cresol, increasing the population of beneficial bacteria and decreasing the pathogenic bacteria [183]. This effect has been also confirmed in an in vivo experiment where cyclophosphamide (CTX)-treated mice were administered with three different polysaccharides from PFSP, obtaining an enhancement in the production of acetic, propionic, and butyric acid, as well as decreasing the ratio of *Firmicutes*/*Bacteroidetes* [180].

### 4.7. Hypouricemic Effect

Hyperuricemia status is caused by the impaired renal excretion of uric acid (UA) which reflects an extracellular fluid supersaturation for UA [226]. Uric acid is the product of the purine metabolism and excreted by the kidneys [227]. The enzymatic degradation of the purine pathway in humans transforms the oxidized hypoxanthine to xanthine, and then to uric acid by enzymes [228]. Hence, the regulative action on the key enzymes in the pathway of uric acid synthesis, 5′-nucleotidase (5′-NT), adenylate deaminase (ADA), and xanthine oxidase (XO), would be essential in their treatment [229,230].

**Figure 3:**
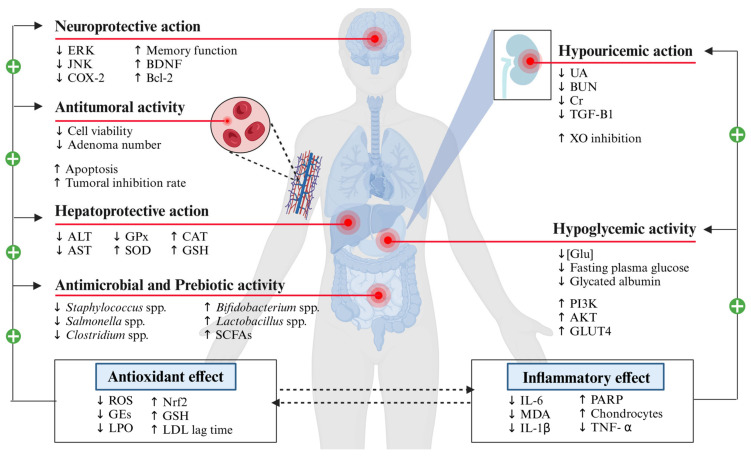
Beneficial effects of *Ipomoea batatas* (L.) Lam. ↑ increase; ↓ decrease.

In this line, some in vivo researchers have studied the hypouricemic effect of PFSP anthocyanins on hyperuricemic mice (Figure 3) [184,185]. Yang et al. [32] reported a significantly decreased level of UA, as well as 5′-NT and XO enzyme activity. The mechanism involved in this action was explained as the insertion of the acyl group of anthocyanins into the hydrophobic region of XO occupying the catalytic center to avoid the entrance of substrate due to the interaction with certain amino acid residues [186,231]. Similar results were reported by Zhang et al. [187] who evaluated the hypouricemic effect of PFSP anthocyanins in hyperuricemia mice showing a reduction in the serum UA level and a regulation of blood urea nitrogen (BUN) and (Cr) levels.

### 4.8. Antimutation and Antitumoral Effect

Cancer is a disease of uncontrolled proliferation by transformed cells subject to evolution by natural selection [232]. The most common cancers are breast, prostate, lung, colon, and bladder cancer which is expected to rise to 29.9 million new cancer cases by 2040 [233]. Several studies conducted in vitro and in vivo reported the antiproliferative and apoptotic effect of components of PFSP and its by-products through the enhancement of their anti-inflammatory and antioxidant properties [234]. Wu et al. [59] isolated three polysaccharides from PFSP and tested its in vitro antitumoral activities confirming that the three of them possessed an inhibitory effect against SGC7901 and SW620 cells in a dose-dependent manner. This effect is related to the structure and spatial conformation of polysaccharides since those with a lower molecular weight and higher sulphate content exhibited stronger antiproliferative activities [235,236]. Another in vitro research project studied the effect of fresh root tubers of the PFSP variety Bhu Krishna and the leaves accession S-1467 on multiple human cancer cell lines, reporting that the cyanidin-rich leaf extracts showed a superior action against colon and cervical cancer cell lines compared to the root [189]. The explanation for this effect is found in the B ring structure of cyanidin which has two hydroxyl groups instead of one in peonidin, predominantly in the leaves [237].

In addition, the antitumoral and antimutation roles of the principal bioactive compound of PFSP, anthocyanins, have been also tested. Guo et al. [188] evaluated the effects of PFSP anthocyanins on acute lymphoblastic leukemia cells (ALL) confirming their antileukemic action through the induction of the p38/c-Myc/CDK1-Cyclin B axis, essential in the progression of the G_2_ phase to the M phase and arresting tumoral DNA synthesis in cells [238,239]. Similar results were found in the administration of PFSP anthocyanins to SPF-grade ICR mice for one month, where the extract inhibited sarcoma S180 cell growth, and the sarcomas were significantly fewer and smaller than in the control mice, achieving an inhibition rate of 69.03% [192].

### 4.9. Other Biological Activities

Over the last few years, more biological activities have been tested, such as cardioprotective, anti-obesity, hypolipidemic, and immunomodulatory effects, as well as lung protection to elucidate the extent to which PFSP bioactive compounds can benefit a healthy status [240,241].

Potential cardioprotective effects of PFSP anthocyanins against low-density lipoprotein (LDL) oxidation in vitro, and atherosclerotic lesions in apolipoprotein E-deficient mice in vivo, reported a potential LDL oxidation protection in vitro and a significantly lower atherosclerotic plaque area, plasma soluble vascular cell adhesion molecule-1 (sVCAM-1), and thiobarbituric acid-reactive substances level than the control mice, suggesting a possible suppression of the development of atherosclerotic lesions [242]. Ju et al. [243] tested the anti-obesity action on high-fat diet-induced obese mice, achieving a reduced body weight and fat accumulation and an improvement in the lipid profile, as well as modulation of the energy intake, reducing the metabolic risk. The immunomodulatory effect of PFSP extracts in immune-deficient induced mice was investigated for 12 weeks, showing the inhibition of lymphadenopathy and the suppression of T- and B-cell proliferation and T helper 1/T helper 2 cytokine imbalance, as well as an increase in SOD and GPx enzyme levels, suggesting an amelioration of immune dysfunction [244]. Dong et al. [245] studied the therapeutic effect of PFSP anthocyanins on *Klebsiella pneumoniae*-infected mice to test the lung protective action. The results showed dampened lung injury, inflammatory responses, and bacterial systemic dissemination in vivo, as well as eliminating pyroptosis and restricting NLRP3 inflammasome activation in alveolar macrophages. According to the data, PFSP bioactive compounds may ameliorate and exert the protective actions mentioned above by modulating antioxidant and anti-inflammatory defense systems [246].

## 5. Food Application

Due to its remarkable health benefits, the use of purple-fleshed sweet potato may be a key opportunity for the food industry in the development of functional food to improve its nutraceutical and functional properties and as a strategy to reduce food waste and improve nutrition [247]. Several studies have considered the incorporation of the *I. batatas* L. purple variety in different food products, especially in bakery products [248,249,250,251,252]. However, none of them have used another PFSP by-product different from flesh and peels. The valorization of PFSP by-products in the form of extracts has become a promising possibility due to the high energetic value of the flesh, as well as the antioxidant properties of the leaves and peels because of their high content of phenolic compounds and anthocyanins (Table 4) [253].

Nowadays, bakery products formulated from refined wheat flour (WF) are dietary basics being consumed in the world despite their being nutritionally poor [254]. Enrichment, fortification, and replacement of some ingredients from staple foods are essential mechanisms to improve the nutrient intakes of the population, in both developed and undeveloped countries, when the staple food is being consumed daily [255]. Worldwide food markets supply a wide spectrum of bakery products, such as various types of bread, biscuits, muffins, cookies, pretzels, or pastries, with protein- and fiber-rich, gluten-free, or sugar-reduced new trends leading a diversification and innovation of the demanded products [256,257,258].

Bread, cookies, muffins, and biscuits are the bakery products studied for the introduction of purple-fleshed sweet potato. Its high anthocyanin and starch content and high-water holding capacity make purple-fleshed sweet potato a great option to incorporate into bread [259]. Several studies have used purple sweet potato flesh and peel in the form of flour to replace wheat, resulting in textural characteristic improvements, and bread with a higher firming rate related to a greater starch–gluten interaction [260,261]. Kweman et al. [262] investigated the physicochemical characteristics and glycemic index of bread made from purple sweet potato flesh flour, starch, and fiber obtained from the solid waste of PFSP starch processing and mixtures in different ratio proportions. The results revealed a higher dietary fiber content than bread made only from wheat flour, indicating its ability to reduce the blood glucose level due to its low glycemic index. Similar results were shown by Zhu et al. [263], who incorporated whole PFSP flour in different mixture ratios with wheat flour, resulting in a 10% replacement PFSP–WF with enhanced functional properties (polyphenols content and in vitro antioxidant activities) and sensory acceptance. However, part of the polyphenols were lost during the steaming stage, suggesting that microencapsulation would be a great strategy to avoid their degradation [264].

Incorporation in pastry products, such as cookies, has also been described [265]. Liu et at. [266] evaluated the quality characteristics and antioxidative activities of cookies using several proportions of PFSP flour, reporting that a concentration between 10 and 20% of PFSP powder was optimum to increase TPC and TA content and antioxidant effects with the best sensory evaluation without altering the sensorial cookie characteristics. Accordingly, another study revealed that PFSP can be incorporated into cookies at up to 20% without affecting the cookie quality and contributing to the deterrence of lipid oxidation after storage at 65 °C for 80 days [267]. Furthermore, Muhammad et al. [268] tested the addition of PSP mashed flesh into crackers, reporting that the PFSP trials were higher in fiber, carbohydrates, protein, anthocyanins, vitamins, and minerals, such as calcium.

Finally, PFSP extracts have also been used to prepare healthier biscuits and muffins [269,270]. Its use in the form of peel extract has also been investigated as an effective way of reducing PFSP by-products from the processing industry and improving functional and nutraceutical properties [271]. The findings suggested that the incorporation of 2% PSP peel powder was optimum for increasing dietary fiber, TPC, TA, and ABTS content, sensory acceptance, and texture preference, since higher concentrations of dry extract would disturb the development of the gluten matrix because of higher amounts of solids and a decreasing amount of gluten–protein content [272]. Nevertheless, limited research studies on PFSP by-product utilization have been performed. According to the outcomes in Table 4, there is a need to investigate the suitable addition form, as well as the optimal percentage that guarantees the health benefits without altering consumer preferences and new encapsulation techniques, which protect the bioactive compound, preventing their degradation and enhancing their stability and bioavailability [192,273,274,275].

**Table 4:** Application of PFSP in bakery products.

Food Product	Formulation	Outcomes	Ref.
**Bread**	Bread made from PSP flesh flour, starch, and fiber from a mixture with a ratio of 75:5:20	Higher dietary fiber content and anthocyanins	[262]
		Lower blood glucose response and glycemic index	
**Bread**	Mixture of whole PFSP (roots and peels) transformed as a freeze-dried flour (5, 10, 20, 30, 40, and 50%), and wheat flour to incorporate into a traditional Chinese steamed bread	Higher TPC and antioxidant activities in the reformulated bread	[263]
		A lower glycemic response	
		Improvement in the overall sensory acceptance at 5–10% levels	
**Cookies and Muffin**	Replacement of wheat flour by the flesh of purple sweet potato in dried form (25, 30, 35, 40, and 45%)	Richness in amino acids and minerals	[270]
		Higher global acceptability	
**Bread**	Mixture of whole PFSP (flesh and peels) transformed as a freeze-dried flour in a proportion of 0, 25, 50, 75, and 100% with wheat flour into a traditional Chinese steamed bread	Decreased viscosity in PFSP breads	[260]
		Increased TPC and antioxidant activity	
		Facilitated pasting wheat flour, decreasing the dough strength	
**Biscuit**	Fresh, flour (using hot air-drying), and paste (by steaming, cooled, and mashed) PFSP was incorporated at 30% with wheat flour in biscuits	Enhancement of the purple color	[276]
		Increased TPC, and TA content and antioxidant activity in a trial formulation	
**Cookies**	Combination of PSP flesh flour (30.93–44.19%) with corn starch in cookies	Improvement in anthocyanin levels	[265]
		Enhancement of the sensory attributes	
**Bread**	4% substitution of the original wheat flour content with PFSP flour in bread	Higher firming rate and greater starch–gluten interaction	[261]
**Cookies**	Substitution of 0, 10, 20, and 30% of PFSP powder	Highest TPC and TA contents and DPPH activity at 30% of addition	[266]
		Increased sensory evaluation in cookies with 10, and 20%	
**Cracker**	Addition of PSP mashed flesh (53%) to crackers	Higher content in fiber, vitamins, and minerals, such as Ca^2+^	[268]
		Elevated TA content	
**Biscuit**	PFSP peel powder was incorporated, at 2, 5, and 10% in biscuits	Dietary fiber increased significantly	[271]
		Enhancement of the TPC, total flavonoids content, and ABTS radical activity	
		Acceptable sensory evaluation at 2% biscuits	
**Biscuit**	PSP flesh flour, fiber, and starch was added in different mixture proportions in biscuits	Biscuits produced from 75% flour and 25% fiber of PSP flour had a good physical and chemical quality	[269]

PSP: purple sweet potato; PFSP: purple-fleshed sweet potato; TPC: total content of phenolic compounds; TA: total anthocyanin; DPPH: 2,2-diphenyl1-1-picrylhydrazyl; ABTS: 2,2′-azino-bis(3-ethylbenzothiazoline-6-sulfonic) acid.

## 6. Conclusions and Future Trends

According to the Food and Agricultural Organization of the United Nations (FAO), food waste contributes to economic losses as well as environmental impact due to the depletion of natural resources such as inputs, water, land, or energy, and market policies result in discarding fit products for human consumption because of aesthetic criteria or due to market prices not being sufficient to cover costs. On the other hand, consumers are concerned about the importance of a healthy diet, generating a change in their food consumption preferences, and they are demanding natural and higher quality products. Additionally, the growing need for a vegetable-based diet where the consumption of animal-based foods is limited for reasons of sustainability, health, and animal welfare has led to the development of new trends in the food industry. Thus, these new trends are focusing more on the revalorization of agricultural by-products, prompting the industry to search for new ingredients that boost efficiency and sustainability. Purple-fleshed sweet potato and its by-products generated during processing have gained increasing attention from companies and consumers due to their richness in bioactive compounds, especially anthocyanins. The flesh, peel, or leaves can be processed into value-added products, which are particularly useful in bakery products with higher added value, such as replacing or substituting traditionally used wheat flour with purple-fleshed sweet potato flour to enhance the nutritional profile. Also, its high leaf protein content could be a good option to enrich pastry products. Hence, the incorporation of PFSP and its by-products could play a dual purpose: (i) integrating the circular economy concept and (ii) providing effects on hepatic, neurological, and glycemic disorders, among others. However, despite the widely recognized health benefit of purple-fleshed sweet potato through in vitro and in vivo studies, the existing literature on the application of its by-products in food is limited. More research is needed to effectively mask the color provided by PFSP, as well as new encapsulation techniques which could provide a promising approach to protect the bioactive compound, preventing its degradation after cooking and enhancing its stability and bioavailability. These aspects are crucial for optimally incorporating PFSP extracts in food products.

In conclusion, *Ipomoea batatas* (L.) Lam is an ideal food in terms of its contents of key bioactive compounds, which are essential for maintaining beneficial health activities that could be utilized for value-added purposes such as food fortification or food additives. For this reason, it is fundamental to harness the significant potential of the purple-fleshed sweet potato and the by-products generated during its processing through an appropriate agro-industrial valorization system.

## Figures and Tables

**Table 2:** Micronutrients and bioactive compounds of diverse botanical parts of PFSP.

Composition		Flesh	Leaves	Peel
Minerals				
Macroelements				
K	(mg/100 g DW)	1200 ^1^	1786.56 ^14^	5572 ^11^
Ca	(mg/100 g DW)	98.20 ^1^	0.26 ^14^	134.30 ^11^
Mg	(mg/100 g DW)	120.40 ^1^	0.16 ^14^	49.7 ^11^
Na	(mg/100 g DW)	55.00 ^1^	0.31 ^14^	38310 ^11^
Microelements				
Zn	(mg/100 g DW)	1.11 ^1^	1.99 ^14^	7.5 ^11^
Se	(mg/100 g DW)	0.23 ^10^	-	-
Fe	(mg/100 g DW)	1.95 ^1^	22.01 ^14^	4.10 ^11^
Mn	(mg/100 g DW)	2.08 ^1^	3.90 ^14^	15 ^11^
Cu	(mg/100 g DW)	0.69 ^1^	0.005 ^14^	0.94 ^11^
Vitamins				
Vitamin B_1_	(mg/100 g DW)	1.89 ^1^	-	-
Vitamin B_2_	(mg/100 g DW)	0.83 ^1^	-	-
Vitamin B_3_	(mg/100 g DW)	2.56 ^1^	-	-
Vitamin C	(mg/100 g DW)	63.40 ^1^	0.30 ^14^	-
	(mg/100 mL DW)	-	-	0.74 ^16^
Vitamin E	(mg/100 g DW)	11.2 ^15^	-	-
No Flavonoids				
Phenolic Compounds				
*m*-Hydroxybenzoic acid	(mg/g DW)	0.11 ^2^	-	-
*P-hydroxybenzoic acid*	(mg/100 g DW)	11.34 ^22^	-	-
*4-Hydroxybenzoic acid*	(μg/g DW)	3.67 ^23^	-	-
Protocatechuic acid-3-glucoside	(mg/g DW)	0.12 ^2^	-	-
Chlorogenic acid	(mg/g DW)	0.34 ^2^	24.90 ^24^	-
Neochlorogenic acid	(mg/100 g DW)	-	158.40 ^24^	-
Cryptochlorogenic acid	(mg/g DW)	0.07 ^2^	2.06 ^24^	-
Isochlorogenic acid	(mg/g DW)	0.20 ^2^	-	-
Caffeic acid	(mg/g DW)	0.17 ^6^	-	0.34 ^25^
	(mg/Kg DW)	-	629.45 ^17^	-
Caffeoyl-hexoside	(mg/100 g DW)	-	-	142 ^25^
5-O-caffeoylquinic acid	(mg/100 g DW)	47	93	245.30 ^25^
3,4-di-O-caffeoylqunic acid	(mg/g DW)	-	3.79 ^24^	0.50 ^25^
3,5-di-O-caffeoylqunic acid	(mg/g DW)	-	4.16 ^24^	6.07 ^25^
Dicaffeoyl quinic acid isomer 1	(mg/g DW)	4.76 ^2^	-	-
	(mg/Kg DW)	-	1051 ^17^	-
Dicaffeoyl quinic acid isomer 2	(mg/g DW)	2.56 ^2^	-	-
	(mg/Kg DW)	-	191.32 ^17^	-
*p*-Coumaric acid	(mg/g DW)	0.72 ^6^	-	-
Trans-*p*-coumaric acid	(mg/100 g DW)	5 04 ^22^	-	-
Coumaroyl-hexoside	(mg/100 g DW)	-	-	60 ^25^
Ferulic acid	(mg/g DW)	0.15 ^2^	-	-
Feruloyl glucose	(mg/g DW)	8.09 ^2^	-	-
Feruloyl sucrose	(mg/g DW)	9.52 ^2^	-	-
Feruloylquinic acid	(mg/100 g DW)	-	-	22.10 ^25^
3-Feruloyl quinic acid	(mg/g DW)	10.62 ^2^	-	-
4-Feruloyl quinic acid	(mg/g DW)	11.77 ^2^	-	-
1,5-Diferuloyl quinic acid	(mg/g DW)	29.56 ^2^	-	-
3-Feruloyl-4-caffeoyl quinic acid	(mg/g DW)	11.77 ^2^	-	-
1-Feruloyl-5-caffeoyl quinic acid	(mg/g DW)	191.57 ^2^	-	-
Salicylic acid	(μg/g DW)	197.88 ^23^	-	-
Protocatechuic acid	(μg/g DW)	74.72 ^23^	-	-
Vanillic acid	(mg/100 g DW)	1.98 ^22^	-	-
Flavonoids				
Anthocyanins				
Cyanidin 3-sophoroside-5-glucoside	(mg PN3GE/kg DW)	312.10 ^7^	3.80 ^7^	-
	(mg/100 g DW)	-	-	50.6 ^25^
Cyanidin 3-(6″-caffeoyl sophoroside)-5-glucoside	(mg/100 g DW)	58.00 ^19^	-	3.6 ^25^
Cyanidin 3-dicaffeoyl sophoroside-5-glucoside	(mg/g DW)	12.20 ^20^	-	-
Cyanidin 3-(6″-feruloyl sophoroside)-5-glucoside	(mg/100 g DW)	95.00 ^19^	-	178.4 ^25^
Cyanidin 3-caffeoyl-p-gydroxybenzoyl sophoroside-5-glucoside	(mg/g DW)	14.80 ^20^	-	-
Cyanidin 3-caffeoyl-feruloyl sophoroside-5-glucoside	(mg/g DW)	16.20 ^20^	-	-
Peonidin 3-sophoroside-5-glucoside	(mg PN3GE/kg DW)	52.90 ^7^	0.80 ^7^	-
	(mg/100 g DW)	-	-	120 ^25^
*p*-hydroxybenzoylated (Cyanidin 3-sophoreside-5-glucoside)	(mg PN3GE/kg DW)	604.60 ^7^	2.70 ^7^	-
Caffeoylated (Cyanidin 3-sophoroside-5-glucoside)	(mg PN3GE/kg DW)	180.10 ^7^	3.30 ^7^	-
*p*-hydroxybenzoylated (Peonidin 3-sophoroside-5-glucoside)	(mg PN3GE/kg DW)	132.80 ^7^	1.00 ^7^	-
Caffeoylated (Peonidin 3-sopheroside-5-glucoside)	(mg PN3GE/kg DW)	46.60 ^7^	1.00 ^7^	-
Feruloylated (Cyanidin 3-sophoroside-5-glucoside)	(mg PN3GE/kg DW)	297 ^7^	1.10 ^7^	-
Cyanidin 3-(6,6′-dicaffeoyl-sophoroside)-5-glucoside	(mg PN3GE/kg DW)	1481.40 ^7^	11.60 ^7^	-
Cyanidin 3-(6,6′-caffeoylphydroxybenzoyl sophoroside)-5-glucoside	(mg PN3GE/kg DW)	5667.90 ^7^	9.80 ^7^	-
Cyanidin 3-(6,6′-caffeoylferuloylsophoroside)-5-glucoside	(mg PN3GE/kg DW)	1877.30 ^7^	1.90 ^7^	-
Peonidin 3-(6″-feruloyl sophoroside)-5-glucoside	(mg/100 g DW)	29 ^19^	-	-
Peonidin 3-(6,6′-dicaffeoylsophoroside)-5-glueoside	(mg PN3GE/kg DW)	381.60 ^7^	2.30 ^7^	-
Peonidin 3-feruloyl-p-hydroxybenzoyl sophoroside-5-glucoside	(mg/g DW)	5.81 ^20^	-	1.12 ^25^
Peonidin 3-(6″, 6‴-diferuloyl sophoroside)-5-glucoside	(mg/g DW)	2.43 ^20^	-	-
Peonidin 3-(6‴-caffeoyl sophoroside)-5-glucoside	(mg/g DW)	2.29 ^20^	-	-
Peonidin 3-feruloyl sophoroside-5-glucoside	(mg/g DW)	7.12 ^20^	-	-
Peonidin 3-dicaffeoyl sophoroside-5-glucoside	(mg/g DW)	57.90 ^20^	-	-
Peonidin 3-caffeoyl sophoroside-5-glucoside	(mg/100 g DW)	275 ^19^	-	-
Peonidin 3-caffeoyl-p-hydroxybenzoyl sophoroside-5-glucoside	(mg/100 g DW)	116 ^19^	-	-
Peonidin 3-(6,6′-caffeoylphydroxybenzoyl sophoroside)-5-glucoside	(mg PN3GE/kg DW)	1620.90 ^7^	1.30 ^7^	-
Peonidin 3-(6,6′-caffeoylferuloylsophoroside)-5-glucoside	(mg PN3GE/kg DW)	344.30 ^7^	1.00 ^7^	-
Peonidin 3-caffeoyl-feruloyl sophoroside-5-glucoside	(mg/g DW)	69.20 ^20^	-	-
Peonidin 3-caffeoyl-p-coumarylsophoroside-5-glucoside	(mg PN3GE/kg DW)	59.30 ^7^	1.10 ^7^	-
Peonidin 3-coumaryl-p-hydroxybenzoyl sophoroside-5-glucoside	(mg/g DW)	1.81 ^20^	-	-
Cyanidin-based anthocyanin	(mg/kg DW)	6964 ^8^	-	-
	(μg/g FW)	-	83.32 ^13^	-
Peonidin-based anthocyanin	(mg/kg DW)	2269 ^8^	-	-
	(μg/g FW)	-	44.01 ^13^	-
Flavonols and Flavones				
Kaempferol	(μg/g DW)	23.38 ^21^	-	-
	(mg/100 g DW)	nd ^9^	60.9 ^9^	nd ^9^
Luteolin	(μg/g DW)	15.17 ^21^	-	-
Myricetin	(μg/g DW)	152.11 ^21^	-	-
	(mg/100 g DW)	42.1 ^9^	36.7 ^9^	20.6 ^9^
Apigenin-6-C-glucoside-8-C-arabinoside	(mg/g DW)	0.82 ^2^	-	-
Naringenin	(mg/g DW)	1.12 ^2^	-	-
Naringenin-glucoside	(mg/g DW)	0.24 ^2^	-	-
Isoquercitin	(mg/100 g DW)	59.9 ^9^	268.3 ^9^	33.9 ^9^
Quercetin-3-galactoside	(mg/g DW)	0.91 ^2^	0.78 ^24^	-
	(mg/Kg DW)	-	455.13 ^17^	-
Quercetin diglucoside	(mg/g DW)	1.02 ^2^	-	-
Isorhamnetin-3-O-glucoside	(mg/g DW)	0.94 ^2^	-	-
Isorhamnetin-3-glucoside 4-rhamnoside	(mg/g DW)	2.12 ^2^	-	-
Epicatechin derivative	(mg/g DW)	0.10 ^2^	-	-
3′-O-Methylepicatechin derivatives	(mg/g DW)	0.05 ^2^	-	-
4′-Methyl-epigallocatechin derivatives	(mg/g DW)	0.09 ^2^	-	-
4′-Methyl-epigallocatechin derivatives	(mg/g DW)	0.02 ^2^	-	-
Carotenoids				
Lutein	(mg/100 g DW)	-	100.22 ^17^	-
	(μg/g DW)	0.28 ^21^	-	-
Zeaxanthin	(mg/100 g DW)	-	33.60 ^17^	-
	(μg/g DW)	0.11 ^21^		
β-Cryptoxanthin	(mg/100 g DW)	0.07 ^18^	-	-
α-Carotene	(μg/g DW)	nd ^21^	-	-
(All *E*)-β-Carotene	(μg/g DW)	1.53 ^21^	-	-
(9Z)-β-Carotene	(μg/g DW)	0.02 ^21^	-	-
(13Z)-β-Carotene	(μg/g DW)	0.28 ^21^	-	-
All-Trans-β-carotene	(mg/100 g DW)	0.30 ^18^	56.94 ^17^	-
Cis-β-carotene	(mg/100 g DW)	-	7.11 ^17^	-
ABTS	(mg AAE/100 g DW)	710 ^9^	5300 ^9^	880 ^9^
DPPH	(mg TE/100 mg DW)	0.77 ^5^	-	0.03 ^11^
	(mg AAE/100 g DW)	330 ^9^	2920 ^9^	410 ^9^
FRAP	(mg TE/100 g DW)	46 ^9^	550 ^9^	47 ^9^
TA	(mg GAE/100 mg DW)	94.80 ^4^	-	-
	(mg CGE/100 g DW)	170 ^9^	1010 ^9^	230 ^9^
TPC	(mg GAE/100 mg DW)	167.40 ^3^	3.68 ^7^	289.01 ^12^

DW: dry weight; FW: fresh weight; FRAP: ferric reducing ability of plasma; TA: total content of anthocyanins; TPC: total content of phenolic compounds; GAE: gallic acid equivalent; DPPH: 2,2-diphenyl-1-picrylhydrazyl; ABTS: 2,2′-azino-bis(3-ethylbenzothiazoline-6-sulfonic) acid; nd: not detected; PN3GE: peonidin 3-glucoside equivalent; AAE: ascorbic acid equivalent; TE: Trolox equivalent; CGE: cyandin-3-glucoside equivalent. ^1^ Mean values of PFSP cultivated in Wanju-Gun rural guidance center [65]; ^2^ Mean values of dry hydroalcoholic purple sweet potato [82]; ^3^ Mean values of the purple-fleshed sweet potato genotype “JNRX12” [83]; ^4^ Mean values of the purple-fleshed sweet potato genotypes “JNRX2” [83]; ^5^ Mean values of the purple-fleshed sweet potato genotypes “Trabuca” [83]; ^6^ Mean values of the purple-fleshed sweet potato genotype “JNRX1” [83]; ^7^ Mean values leaves or flesh of PFSP “P40” [74]; ^8^ Mean values of the purple-fleshed sweet potato variety “Gyebone108” [84]; ^9^ Mean values of the PFSP variety “Yuzi No. 7” [85]; ^10^ Mean values of the PFSP variety “Jishu No.2” [63]; ^11^ Mean value of a PFSP variety bought from the King’s market, Akure, Ondo State, Nigeria [86]; ^12^ Mean value of *Ipomoea batatas* Lam *cv. Anggun 1* harvested in Malaysia [87]; ^13^ Mean value of the PFSP cultivar Fushu No. 317 [74]; ^14^ Mean value of PFSP from community gardens in Koya Koso Jayapura [88]; ^15^ Mean value of PFSP accession from a farmer’s field for planting material [89]; ^16^ Mean value of *Ipomoea batatas* Lam cv. Anggun 1 [90]; ^17^ Mean value of PFSP leaves of Purple-purple X VS-8 WAP, and Purple-purple X TMS-16 WAP samples [91]; ^18^ Mean value of the PFSP variety *Yeonjami* [92]; ^19^ Mean value of the PFSP variety P40 [93]; ^20^ Mean value of *Ipomoea batatas* L. cultivar *Eshu* No. 8 [33]; ^21^ Mean value of the PFSP cultivar Sinjami [94]; ^22^ Mean value of the PFSP cultivar *Dphi potato* 1, *Wuxi*, and *Jiangsu* [95]; ^23^ Mean value of the PFSP cv. *Yeonjami* [96]; ^24^ Mean value of a purple sweet potato cultivar [97]; ^25^ Mean value of the PFSP genotype Ea3-1 [75].

## References

[1] Mwanga R.O.M., Andrade M.I., Carey E.E., Low J.W., Craig Yencho G., Grüneberg W.J. (2017). Sweetpotato (*Ipomoea batatas* L.). Genetic Improvement of Tropical Crops.

[2] Drapal M., Rossel G., Heider B., Fraser P.D. (2019). Metabolic Diversity in Sweet Potato (*Ipomoea batatas*, Lam.) Leaves and Storage Roots. Hortic. Res..

[3] Sullivan D. (2016). Sweet Potato: Production, Nutritional Properties and Diseases.

[4] Prohens J., Alvarez J.B., Khoury C.K. (2015). Distributions, Ex Situ Conservation Priorities, and Genetic Resource Potential of Crop Wild Relatives of Sweetpotato [*Ipomoea batatas* (L.) Lam., *I. Series Batatas*]. Front. Plant Sci..

[5] Jiang T., Ye S., Liao W., Wu M., He J., Mateus N., Oliveira H. (2022). The Botanical Profile, Phytochemistry, Biological Activities and Protected-Delivery Systems for Purple Sweet Potato (*Ipomoea batatas* (L.) Lam.): An up-to-Date Review. Food Res. Int..

[6] Hall M.R., Phatak S.C. (1993). Sweet Potato: *Ipomoea batatas* (L.) Lam. Genetic Improvement of Vegetable Crops.

[7] Kays S.J. (2005). Sweetpotato Production Worldwide: Assessment, Trends and the Future. Acta Hortic..

[8] Food and Agriculture Organization of the United Nations FAOSTAT. https://www.fao.org/faostat/en/#data/QCL.

[9] Ministerio de Agricultura Pesca y Alimentación MAPA. https://www.mapa.gob.es/es/estadistica/temas/publicaciones/anuario-de-estadistica/2022/default.aspx?parte=3&capitulo=07&grupo=3.

[10] Mohanraj R., Sivasankar S. (2014). Sweet Potato (*Ipomoea batatas* [L.] Lam)—A Valuable Medicinal Food: A Review. J Med. Food.

[11] Nedunchezhiyan M., Gangadharan B., Jata S.K. (2012). Sweet Potato Agronomy. Fruit Veg. Cereal Sci. Biotechnol..

[12] Doganay K.H., Kurunc A., Dincer C. (2023). Salinity Stress Effects on the Growth Yield and Quality Performance of Two Sweet Potato Varieties. ACS Agric. Sci. Technol..

[13] Pushpalatha R., Gangadharan B. (2024). Climate Resilience, Yield and Geographical Suitability of Sweet Potato under the Changing Climate: A Review. Nat. Resour. Forum.

[14] Bovell-Benjamin A.C. (2007). Sweet Potato: A Review of Its Past, Present, and Future Role in Human Nutrition. Adv. Food Nutr. Res..

[15] Tan W., Guo X., Wang Z., Zhang R., Tang C., Jiang B., Jia R., Deng Y., Yang S., Chen J. (2024). Metabolic Profiles and Morphological Characteristics of Leaf Tips among Different Sweet Potato (*Ipomoea batatas* Lam.) Varieties. J. Integr. Agric..

[16] Cui L., Liu C.-Q., Li D.-J., Song J.-F. (2011). Effect of Processing on Taste Quality and Health-Relevant Functionality of Sweet Potato Tips. Agric. Sci. China.

[17] Richardson M.L., Arlotta C.G. (2023). Growing Sweet Potatoes [*Ipomoea batatas* (L.) Lam.)] for Their Greens and the Impact on Storage Roots. J. Hortic. Sci..

[18] Hamda, Ahmad M.Q., Saleem A., Yan H., Li Q. (2024). Biofortified Sweet Potato—An Ideal Source of Mitigating Hidden Hunger. Biofortification Grain Veg. Crops Mol. Breed. Approaches.

[19] Akoetey W., Britain M.M., Morawicki R.O. (2017). Potential Use of Byproducts from Cultivation and Processing of Sweet Potatoes. Ciência Rural..

[20] Woolfe A.J. (1992). Sweet Potato: An Untapped Food Resource.

[21] Nieto G., Bañón S., Garrido M. (2012). Administration of distillate thyme leaves into the diet of Segureña ewes: Effect on lamb meat quality. Animal.

[22] Alam M.K. (2021). A Comprehensive Review of Sweet Potato (*Ipomoea batatas* [L.] Lam): Revisiting the Associated Health Benefits. Trends Food Sci. Technol..

[23] Terahara N., Konczak I., Ono H., Yoshimoto M., Yamakawa O. (2004). Characterization of Acylated Anthocyanins in Callus Induced from Storage Root of Purple-Fleshed Sweet Potato, *Ipomoea batatas* L.. J. Biomed. Biotechnol..

[24] Philpott M., Gould K.S., Lim C., Ferguson L.R. (2004). In Situ and In Vitro Antioxidant Activity of Sweetpotato Anthocyanins. J. Agric. Food Chem..

[25] Sanoussi A.F., Dansi A., Ahissou H., Adebowale A., Sanni L.O., Orobiyi A., Dansi M., Azokpota P., Sanni A. (2016). Possibilities of Sweet Potato [*Ipomoea batatas* (L.) Lam] Value Chain Upgrading as Revealed by Physico-Chemical Composition of Ten Elites Landraces of Benin. Afr. J. Biotechnol..

[26] Teow C.C., Den Truong V., McFeeters R.F., Thompson R.L., Pecota K.V., Yencho G.C. (2007). Antioxidant Activities, Phenolic and β-Carotene Contents of Sweet Potato Genotypes with Varying Flesh Colours. Food Chem..

[27] Li A., Xiao R., He S., An X., He Y., Wang C., Yin S., Wang B., Shi X., He J. (2019). Research Advances of Purple Sweet Potato Anthocyanins: Extraction, Identification, Stability, Bioactivity, Application, and Biotransformation. Molecules.

[28] Huang G.J., Sheu M.J., Chang Y.S., Lu T.L., Chang H.Y., Huang S.S., Lin Y.H. (2008). Isolation and Characterisation of Invertase Inhibitor from Sweet Potato Storage Roots. J. Sci. Food Agric..

[29] Zhao J.G., Yan Q.Q., Lu L.Z., Zhang Y.Q. (2013). In Vivo Antioxidant, Hypoglycemic, and Anti-Tumor Activities of Anthocyanin Extracts from Purple Sweet Potato. Nutr. Res. Pract..

[30] Yoshimoto M., Okuno S., Yamaguchi M., Yamakawa O. (2001). Antimutagenicity of Deacylated Anthocyanins in Purple-Fleshed Sweetpotato. Biosci. Biotechnol. Biochem..

[31] Wu Q., Qu H., Jia J., Kuang C., Wen Y., Yan H., Gui Z. (2015). Characterization, Antioxidant and Antitumor Activities of Polysaccharides from Purple Sweet Potato. Carbohydr. Polym..

[32] Yang Y., Zhang Z.-C., Zhou Q., Yan J.-X., Zhang J.-L., Su G.-H. (2020). Hypouricemic Effect in Hyperuricemic Mice and Xanthine Oxidase Inhibitory Mechanism of Dietary Anthocyanins from Purple Sweet Potato (*Ipomoea batatas* L.). J. Funct. Foods.

[33] Wang L., Zhao Y., Zhou Q., Luo C.L., Deng A.P., Zhang Z.C., Zhang J.L. (2017). Characterization and Hepatoprotective Activity of Anthocyanins from Purple Sweet Potato (*Ipomoea batatas* L. *Cultivar Eshu* No. 8). J. Food Drug Anal..

[34] Zhuang J., Lu J., Wang X., Wang X., Hu W., Hong F., Zhao X.-X., Zheng Y.-L. (2019). Purple Sweet Potato Color Protects against High-Fat Diet-Induced Cognitive Deficits through AMPK-Mediated Autophagy in Mouse Hippocampus. J. Nutr. Biochem..

[35] De Castro Vendrame L.P., de Castro e Melo R.A., da Silva G.O., Vargas P.F., Leonel M. (2023). Sweet Potato (*Ipomoea batatas* L. Lam.) Cultivation and Potentialities. Var. Landraces Cult. Pract. Tradit. Uses.

[36] Guo Z., Zhao B., Li H., Miao S., Zheng B. (2019). Optimization of Ultrasound-Microwave Synergistic Extraction of Prebiotic Oligosaccharides from Sweet Potatoes (*Ipomoea batatas* L.). Innov. Food Sci. Emerg. Technol..

[37] Rodríguez-Mena A., Ochoa-Martínez L.A., González-Herrera S.M., Rutiaga-Quiñones O.M., González-Laredo R.F., Olmedilla-Alonso B., Vega-Maturino S. (2023). Coloring Potential of Anthocyanins from Purple Sweet Potato Paste: Ultrasound-Assisted Extraction, Enzymatic Activity, Color and Its Application in Ice Pops. Food Chem. Adv..

[38] Liu W., Yang C., Zhou C., Wen Z., Dong X. (2019). An Improved Microwave-Assisted Extraction of Anthocyanins from Purple Sweet Potato in Favor of Subsequent Comprehensive Utilization of Pomace. Food Bioprod. Process..

[39] Zhang L., Zhao L., Bian X., Guo K., Zhou L., Wei C. (2018). Characterization and Comparative Study of Starches from Seven Purple Sweet Potatoes. Food Hydrocoll..

[40] Gajanayake B., Raja Reddy K., Shankle M.W., Arancibia R.A., Villordon A.O., Arancibia R.A., Shankle M.W. (2014). Quantifying Storage Root Initiation, Growth, and Developmental Responses of Sweetpotato to Early Season Temperature. Agron. J..

[41] Hossain M.M., Rahim M.A., Moutosi H.N., Das L. (2022). Evaluation of the Growth, Storage Root Yield, Proximate Composition, and Mineral Content of Colored Sweet Potato Genotypes. J. Agric. Food Res..

[42] Ivane N.M.A., Wang W., Ma Q., Wang J., Sun J. (2024). Harnessing the Health Benefits of Purple and Yellow-Fleshed Sweet Potatoes: Phytochemical Composition, Stabilization Methods, and Industrial Utilization—A Review. Food Chem. X.

[43] U.S. Department of Agriculture (2021). [Historical Record]: Purple Sweet Potatoes.

[44] Martínez-Zamora L., Penalver R., Ros G., Nieto G. (2021). Substitution of synthetic nitrates and antioxidants by spices, fruits and vegetables in clean label Spanishchorizo. Food Res. Int..

[45] Fan H., Liang D., Fu F., Xu M., Li Z., Suo B., Ai Z. (2022). Processing Suitability of Different Varieties of Sweet Potatoes Cooked with Different Methods. J. Food Process. Preserv..

[46] Lebot V. (2017). Rapid Quantitative Determination of Maltose and Total Sugars in Sweet Potato (*Ipomoea batatas* L. [Lam.]) Varieties Using HPTLC. J. Food Sci. Technol..

[47] Wang H., Yang Q., Ferdinand U., Gong X., Qu Y., Gao W., Ivanistau A., Feng B., Liu M. (2020). Isolation and Characterization of Starch from Light Yellow, Orange, and Purple Sweet Potatoes. Int. J. Biol. Macromol..

[48] Guo K., Liu T., Xu A., Zhang L., Bian X., Wei C. (2019). Structural and Functional Properties of Starches from Root Tubers of White, Yellow, and Purple Sweet Potatoes. Food Hydrocoll..

[49] Sun H., Fan J., Tian Z., Ma L., Meng Y., Yang Z., Zeng X., Liu X., Kang L., Nan X. (2022). Effects of Treatment Methods on the Formation of Resistant Starch in Purple Sweet Potato. Food Chem..

[50] Yong H., Wang X., Sun J., Fang Y., Liu J., Jin C. (2018). Comparison of the Structural Characterization and Physicochemical Properties of Starches from Seven Purple Sweet Potato Varieties Cultivated in China. Int. J. Biol. Macromol..

[51] Wang S., Pan D., Lv X., Song X., Qiu Z., Huang C., Huang R., Chen W. (2016). Proteomic Approach Reveals That Starch Degradation Contributes to Anthocyanin Accumulation in Tuberous Root of Purple Sweet Potato. J. Proteom..

[52] Stribling P., Ibrahim F. (2023). Dietary Fibre Definition Revisited—The Case of Low Molecular Weight Carbohydrates. Clin. Nutr. ESPEN.

[53] Han X., Ma Y., Ding S., Fang J., Liu G. (2023). Regulation of Dietary Fiber on Intestinal Microorganisms and Its Effects on Animal Health. Anim. Nutr..

[54] Zhu S., Sun H., Mu T., Li Q., Richel A. (2023). Preparation of Cellulose Nanocrystals from Purple Sweet Potato Peels by Ultrasound-Assisted Maleic Acid Hydrolysis. Food Chem..

[55] Kurnianingsih N., Ratnawati R., Nazwar T.A., Ali M., Fatchiyah F. (2020). A Comparative Study on Nutritional Value of Purple Sweet Potatoes from West Java and Central Java, Indonesia. J. Phys. Conf. Ser..

[56] Kurnianingsih N., Ratnawati R., Fatchiyah F., Barlianto W., Ali M.M., Safitri A., Suyanto E. (2019). The Difference of Amino Acid Profiling from Two Morphological Purple Sweet Potatoes from Kawi Mountain Cultivars, East Java, Indonesia. J. Phys. Conf. Ser..

[57] Jiang T., Shuai X., Li J., Yang N., Deng L., Li S., He Y., Guo H., Li Y., He J. (2020). Protein-Bound Anthocyanin Compounds of Purple Sweet Potato Ameliorate Hyperglycemia by Regulating Hepatic Glucose Metabolism in High-Fat Diet/Streptozotocin-Induced Diabetic Mice. J. Agric. Food Chem..

[58] Tang C.C., Ameen A., Fang B.P., Liao M.H., Chen J.Y., Huang L.F., Zou H.D., Wang Z.Y. (2021). Nutritional Composition and Health Benefits of Leaf-Vegetable Sweet Potato in South China. J. Food Compos. Anal..

[59] Bañón S., Díaz P., Nieto G., Castillo M., Álvarez D. (2008). Modelling the Yield and Texture of Comminuted Pork Products Using Color and Temperature. Effect of Fat/Lean Ratio and Starch. Meat Sci..

[60] Curayag Q.A.L., Dizon E.I., Hurtada W.A. (2019). Antioxidant Activity, Chemical and Nutritional Properties of Raw and Processed Purple-Fleshed Sweet Potato (*Ipomoea batatas* Lam.). Cogent. Food Agric..

[61] Ji H., Zhang H., Li H., Li Y. (2015). Analysis on the Nutrition Composition and Antioxidant Activity of Different Types of Sweet Potato Cultivars. Food Nutr. Sci..

[62] Munekata P.E.S., Pateiro M., Domínguez R., Nieto G., Kumar M., Dhama K., Lorenzo J.M. (2023). Bioactive compounds from fruits as preservatives. Foods.

[63] Sinanoglou J., Xu M., Li J., Yin J., Wu M., Zhou W., Yang X., Zhang R., He J. (2024). Color and Nutritional Analysis of Ten Different Purple Sweet Potato Varieties Cultivated in China via Principal Component Analysis and Cluster Analysis. Foods.

[64] Lim S., Xu J., Kim J., Chen T.Y., Su X., Standard J., Carey E., Griffin J., Herndon B., Katz B. (2013). Role of Anthocyanin-Enriched Purple-Fleshed Sweet Potato P40 in Colorectal Cancer Prevention. Mol. Nutr. Food Res..

[65] Kim S.Y., Ryu C.H. (1995). Studies on the Nutritional Components of Purple Sweet Potato (*Ipomoea batatas*). Korean J. Food Sci. Technol..

[66] Martínez L., Ros G., Nieto G. (2018). Fe, Zn and Se bioavailability in chicken meat emulsions enriched with minerals, hydroxytyrosol and extra virgin olive oil as measured by Caco-2 cell model. Nutrients.

[67] Grace M.H., Yousef G.G., Gustafson S.J., Truong V.D., Yencho G.C., Lila M.A. (2014). Phytochemical Changes in Phenolics, Anthocyanins, Ascorbic Acid, and Carotenoids Associated with Sweetpotato Storage and Impacts on Bioactive Properties. Food Chem..

[68] Enaru B., Drețcanu G., Pop T.D., Stǎnilǎ A., Diaconeasa Z. (2021). Anthocyanins: Factors Affecting Their Stability and Degradation. Antioxidants.

[69] Chen C.C., Lin C., Chen M.H., Chiang P.Y. (2019). Stability and Quality of Anthocyanin in Purple Sweet Potato Extracts. Foods.

[70] Hu Y., Deng L., Chen J., Zhou S., Liu S., Fu Y., Yang C., Liao Z., Chen M. (2016). An Analytical Pipeline to Compare and Characterise the Anthocyanin Antioxidant Activities of Purple Sweet Potato Cultivars. Food Chem..

[71] Luo X., Wang R., Wang J., Li Y., Luo H., Chen S., Zeng X., Han Z. (2022). Acylation of Anthocyanins and Their Applications in the Food Industry: Mechanisms and Recent Research Advances. Foods.

[72] Putu Eka Widyadharma I., Purwata T.E., Suprapta D.N., Raka Sudewi A.A. (2020). Anthocyanin Derived from Purple Sweet Potato Water Extracts Ameliorated Oxidative Stress, Inflammation, Mechanical Allodynia, and Cold Allodynia among Chronic Constriction Injury-Induced Neuropathic Pain in Rats. Open Access Maced. J. Med. Sci..

[73] Islam M.S., Yoshimoto M., Terahara N., Yamakawa O. (2002). Anthocyanin Compositions in Sweetpotato (*Ipomoea batatas* L.) Leaves. Biosci. Biotechnol. Biochem..

[74] Li G.L., Lin Z., Zhang H., Liu Z., Xu Y., Xu G., Li H., Ji R., Luo W., Qiu Y. (2019). Anthocyanin Accumulation in the Leaves of the Purple Sweet Potato (*Ipomoea batatas* L.) Cultivars. Molecules.

[75] Zhu F., Cai Y.Z., Yang X., Ke J., Corke H. (2010). Anthocyanins, Hydroxycinnamic Acid Derivatives, and Antioxidant Activity in Roots of Different Chinese Purple-Fleshed Sweetpotato Genotypes. J. Agric. Food Chem..

[76] Su X., Griffin J., Xu J., Ouyang P., Zhao Z., Wang W. (2019). Identification and Quantification of Anthocyanins in Purple-Fleshed Sweet Potato Leaves. Heliyon.

[77] Mano H., Ogasawara F., Sato K., Higo H., Minobe Y. (2007). Isolation of a Regulatory Gene of Anthocyanin Biosynthesis in Tuberous Roots of Purple-Fleshed Sweet Potato. Plant Physiol..

[78] Fenger J.A., Roux H., Robbins R.J., Collins T.M., Dangles O. (2021). The Influence of Phenolic Acyl Groups on the Color of Purple Sweet Potato Anthocyanins and Their Metal Complexes. Dye. Pigment..

[79] Jiang T., Mao Y., Sui L., Yang N., Li S., Zhu Z., Wang C., Yin S., He J., He Y. (2019). Degradation of Anthocyanins and Polymeric Color Formation during Heat Treatment of Purple Sweet Potato Extract at Different PH. Food Chem..

[80] Fan L., Wang Y., Xie P., Zhang L., Li Y., Zhou J. (2019). Copigmentation Effects of Phenolics on Color Enhancement and Stability of Blackberry Wine Residue Anthocyanins: Chromaticity, Kinetics and Structural Simulation. Food Chem..

[81] Qian B.J., Liu J.H., Zhao S.J., Cai J.X., Jing P. (2017). The Effects of Gallic/Ferulic/Caffeic Acids on Colour Intensification and Anthocyanin Stability. Food Chem..

[82] Rodriguez L., Muñoz-Bernal Ó.A., Fuentes E., Alvarez-Parrilla E., Palomo I., Wall-Medrano A. (2024). Phenolic Profile, Cheminformatics, and Antiplatelet Aggregation Activity of Orange and Purple Sweet Potato (*Ipomoea batatas* L.) Storage Roots. Food Chem..

[83] Basílio L.S.P., Nunes A., Minatel I.O., Diamante M.S., Di Lázaro C.B., Silva A.C.A.F.E., Vargas P.F., Vianello F., Maraschin M., Lima G.P.P. (2024). The Phytochemical Profile and Antioxidant Activity of Thermally Processed Colorful Sweet Potatoes. Horticulturae.

[84] Jang H.H., Kim H.W., Kim S.Y., Kim S.M., Kim J.B., Lee Y.M. (2019). In Vitro and in Vivo Hypoglycemic Effects of Cyanidin 3-Caffeoyl-p-Hydroxybenzoylsophoroside-5-Glucoside, an Anthocyanin Isolated from Purple-Fleshed Sweet Potato. Food Chem..

[85] Makori S.I., Mu T.H., Sun H.N. (2020). Total Polyphenol Content, Antioxidant Activity, and Individual Phenolic Composition of Different Edible Parts of 4 Sweet Potato Cultivars. Nat. Prod. Commun..

[86] Salawu S.O., Udi E., Akindahunsi A.A., Boligon A.A., Athayde M.L. (2015). Antioxidant Potential, Phenolic Profile and Nutrient Composition of Flesh and Peels from Nigerian White and Purple Skinned Sweet Potato (*Ipomea batatas* L.). Pelagia Res. Libr. Asian J. Plant Sci. Res..

[87] Shamsudin R., Shaari N., Noor M.Z.M., Azmi N.S., Hashim N. (2022). Evaluation of Phytochemical and Mineral Composition of Malaysia’s Purple-Flesh Sweet Potato. Pertanika J. Sci. Technol..

[88] Ranteallo Y., Ahmad M., Syam A., Nilawati A. (2023). Identification and Quantification of Minerals and Vitamins of Purple Sweet Potato (*Ipomoea batatas*) Leave. IOP Conf. Ser. Earth Environ. Sci..

[89] Izalin Mohamad Zahari N., Karuppan J., Shah Shaari E., Mohamad K., Othman R., Yaacob Y. (2016). Quality Attributes of Different Purple Sweet Potato Variety and Sensory Evaluation of Purple Sweet Potato Straight Drink. Regional Conference on Science, Technology and Social Sciences (RCSTSS 2014).

[90] Shaari N., Shamsudin R., Nor M.Z.M., Hashim N. (2021). Quality Attributes of Malaysia Purple-Fleshed Sweet Potato at Different Peel Condition. Agronomy.

[91] Tshilongo L., Mianda S.M., Seke F., Laurie S.M., Sivakumar D. (2024). Influence of Harvesting Stages on Phytonutrients and Antioxidant Properties of Leaves of Five Purple-Fleshed Sweet Potato (*Ipomoea batatas*) Genotypes. Foods.

[92] Kim H.J., Park W.S., Bae J.Y., Kang S.Y., Yang M.H., Lee S., Lee H.S., Kwak S.S., Ahn M.J. (2015). Variations in the Carotenoid and Anthocyanin Contents of Korean Cultural Varieties and Home-Processed Sweet Potatoes. J. Food Compos. Anal..

[93] Xu J., Su X., Lim S., Griffin J., Carey E., Katz B., Tomich J., Smith J.S., Wang W. (2015). Characterisation and Stability of Anthocyanins in Purple-Fleshed Sweet Potato P40. Food Chem..

[94] Park S.Y., Lee S.Y., Yang J.W., Lee J.S., Oh S.D., Oh S., Lee S.M., Lim M.H., Park S.K., Jang J.S. (2016). Comparative Analysis of Phytochemicals and Polar Metabolites from Colored Sweet Potato (*Ipomoea batatas* L.) Tubers. Food Sci. Biotechnol..

[95] Chen Y., Xu Y., Cao Y., Fang K., Xia W., Jiang Q. (2017). Combined Effect of Microwave and Steam Cooking on Phytochemical Compounds and Antioxidant Activity of Purple Sweet Potatoes. Food Sci. Technol. Res..

[96] Kim M.Y., Lee B.W., Lee H.U., Lee Y.Y., Kim M.H., Lee J.Y., Lee B.K., Woo K.S., Kim H.J. (2019). Phenolic Compounds and Antioxidant Activity in Sweet Potato after Heat Treatment. J. Sci. Food Agric..

[97] Krochmal-Marczak B., Cebulak T., Kapusta I., Oszmiański J., Kaszuba J., Zurek N. (2020). The Content of Phenolic Acids and Flavonols in the Leaves of Nine Varieties of Sweet Potatoes (*Ipomoea batatas* L.) Depending on Their Development, Grown in Central Europe. Molecules.

[98] Jang Y., Koh E. (2019). Antioxidant Content and Activity in Leaves and Petioles of Six Sweet Potato (*Ipomoea batatas* L.) and Antioxidant Properties of Blanched Leaves. Food Sci. Biotechnol..

[99] Ooi S.F., Sukri S.A.M., Zakaria N.N.A., Harith Z.T. (2021). Carotenoids, Phenolics and Antioxidant Properties of Different Sweet Potatoes (*Ipomoea batatas*) Varieties. IOP Conf. Ser. Earth Environ. Sci..

[100] Rumbaoa R.G.O., Cornago D.F., Geronimo I.M. (2009). Phenolic Content and Antioxidant Capacity of Philippine Sweet Potato (*Ipomoea batatas*) Varieties. Food Chem..

[101] Huang Y.C., Chang Y.H., Shao Y.Y. (2006). Effects of Genotype and Treatment on the Antioxidant Activity of Sweet Potato in Taiwan. Food Chem..

[102] Jia R., Tang C., Chen J., Zhang X., Wang Z. (2022). Total Phenolics and Anthocyanins Contents and Antioxidant Activity in Four Different Aerial Parts of Leafy Sweet Potato (*Ipomoea batatas* L.). Molecules.

[103] Padda M.S., Picha D.H. (2007). Antioxidant Activity and Phenolic Composition in “beauregard” Sweetpotato Are Affected by Root Size and Leaf Age. J. Am. Soc. Hortic. Sci..

[104] Zeroual A., Sakar E.H., Mahjoubi F., Chaouch M., Chaqroune A., Taleb M. (2022). Effects of Extraction Technique and Solvent on Phytochemicals, Antioxidant, and Antimicrobial Activities of Cultivated and Wild Rosemary (*Rosmarinus Officinalis* L.) from Taounate Region (Northern Morocco). Biointerface Res. Appl. Chem..

[105] Wang A., Li R., Ren L., Gao X., Zhang Y., Ma Z., Ma D., Luo Y. (2018). A Comparative Metabolomics Study of Flavonoids in Sweet Potato with Different Flesh Colors (*Ipomoea batatas* (L.) Lam). Food Chem..

[106] Terahara N. (2015). Flavonoids in Foods: A Review. Nat. Prod. Commun..

[107] Sun W., Zhang M., Chen H., Zheng D., Fang Z. (2016). Effects of Deodorization on the Physicochemical Index and Volatile Compounds of Purple Sweet Potato Anthocyanins (PSPAs). LWT.

[108] Bechoff A. (2010). Investigating Carotenoid Loss after Drying and Storage of Orange-Fleshed Sweet Potato. Ph.D. Thesis.

[109] Ayuso P., Quizhpe J., Rosell M.d.l.Á., Peñalver R., Nieto G. (2024). Bioactive Compounds, Health Benefits and Food Applications of Artichoke (*Cynara Scolymus* L.) and Artichoke By-Products: A Review. Appl. Sci..

[110] Gröber U., Schmidt J., Kisters K. (2015). Magnesium in Prevention and Therapy. Nutrients.

[111] Buffington M.A., Abreo K. (2016). Hyponatremia: A Review. J. Intensive Care Med..

[112] Robertson J.I.S. (1984). Diuretics, Potassium Depletion and the Risk of Arrhythmias. Eur. Heart J..

[113] Nieto G., Martínez L., Castillo J., Ros G. (2017). Effect of hydroxytyrosol, walnut and olive oil on nutritional profile of Low-Fat Chicken Frankfurters. Eur. J. Lipid Sci. Technol..

[114] Ikanone C.E.O., Oyekan P.O. (2014). Effect of Boiling and Frying on the Total Carbohydrate, Vitamin C and Mineral Contents of Irish (Solanun Tuberosum) and Sweet (*Ipomea batatas*) Potato Tubers. Niger. Food J..

[115] Sir Elkhatim K.A., Elagib R.A.A., Hassan A.B. (2018). Content of Phenolic Compounds and Vitamin C and Antioxidant Activity in Wasted Parts of Sudanese Citrus Fruits. Food Sci. Nutr..

[116] Valdés F. (2006). Vitamina C. Actas Dermosifiliogr..

[117] Guclu G., Dagli M.M., Aksay O., Keskin M., Kelebek H., Selli S. (2023). Comparative Elucidation on the Phenolic Fingerprint, Sugars and Antioxidant Activity of White, Orange and Purple-Fleshed Sweet Potatoes (*Ipomoea batatas* L.) as Affected by Different Cooking Methods. Heliyon.

[118] Lv X., Mu J., Wang W., Liu Y., Lu X., Sun J., Wang J., Ma Q. (2022). Effects and Mechanism of Natural Phenolic Acids/Fatty Acids on Copigmentation of Purple Sweet Potato Anthocyanins. Curr. Res. Food Sci..

[119] Schieber M., Chandel N.S. (2014). ROS Function in Redox Signaling and Oxidative Stress. Curr. Biol..

[120] Valko M., Leibfritz D., Moncol J., Cronin M.T.D., Mazur M., Telser J. (2007). Free Radicals and Antioxidants in Normal Physiological Functions and Human Disease. Int. J. Biochem. Cell Biol..

[121] Chintha P., Sarkar D., Pecota K., Dogramaci M., Hatterman-Valenti H., Shetty K. (2023). Phenolic Bioactive-Linked Antioxidant, Anti-Hyperglycemic, and Antihypertensive Properties of Sweet Potato Cultivars with Different Flesh Color. Hortic. Environ. Biotechnol..

[122] Oki T., Sato M., Yoshinaga M., Sakai T., Sugawara T., Terahara N., Suda I. (2009). 1, 1-Diphenyl-2-Picrylhydrazyl Radical-Scavenging Capacity and Oxygen Radical Absorbance Capacity of Sweet Potato Cultivars with Various Flesh Colors. J. Jpn. Soc. Food Sci. Technol.—Nippon. Shokuhin Kagaku Kogaku Kaishi.

[123] Esatbeyoglu T., Rodríguez-Werner M., Schlösser A., Winterhalter P., Rimbach G. (2017). Fractionation, Enzyme Inhibitory and Cellular Antioxidant Activity of Bioactives from Purple Sweet Potato (*Ipomoea batatas*). Food Chem..

[124] Ryan M.J., Jackson J.R., Hao Y., Leonard S.S., Alway S.E. (2011). Inhibition of Xanthine Oxidase Reduces Oxidative Stress and Improves Skeletal Muscle Function in Response to Electrically Stimulated Isometric Contractions in Aged Mice. Free Radic. Biol. Med..

[125] He F., Ru X., Wen T. (2020). Molecular Sciences NRF2, a Transcription Factor for Stress Response and Beyond. Int. J. Mol. Sci..

[126] Ye J., Xiangjun A.E., Ae M., Ae C.Y., Wang C. (2010). Effect of Purple Sweet Potato Anthocyanins on B-Amyloid-Mediated PC-12 Cells Death by Inhibition of Oxidative Stress. Neurochem. Res..

[127] Insanu M., Amalia R., Fidrianny I. (2022). Potential Antioxidative Activity of Waste Product of Purple Sweet Potato (*Ipomoea batatas* Lam). Pak. J. Biol. Sci..

[128] Chen C.-M., Lin Y.-L., Oliver C.-Y., Phd C., Hsu C.-Y., Shieh M.-J., Liu J.-F., Phd R.D. (2008). Consumption of Purple Sweet Potato Leaves Decreases Lipid Peroxidation and DNA Damage in Humans. Asia Pac. J. Clin. Nutr..

[129] Chang W.-H., Chen Msc C.-M., Hu S.-P., Kan N.-W., Chiu C.-C., Liu J.-F. (2007). Effect of Purple Sweet Potato Leaf Consumption on the Modulation of the Antioxidative Status in Basketball Players during Training. Asia Pac. J. Clin. Nutr..

[130] Zhang Y., Niu F., Sun J., Xu F., Yue R. (2015). Purple Sweet Potato (*Ipomoea batatas* L.) Color Alleviates High-Fat-Diet-Induced Obesity in SD Rat by Mediating Leptin’s Effect and Attenuating Oxidative Stress. Food Sci. Biotechnol.

[131] Chang W.-H., Hu S.-P., Huang Y.-F., Yeh T.-S., Liu J.-F. (2010). Effect of Purple Sweet Potato Leaves Consumption on Exercise-Induced Oxidative Stress and IL-6 and HSP72 Levels. J. Appl. Physiol..

[132] Kano M., Takayanagi T., Harada K., Makino K., Ishikawa F. (2005). Antioxidative Activity of Anthocyanins from Purple Sweet Potato, *Ipomoera batatas* Cultivar Ayamurasaki. Biosci. Biotechnol. Biochem..

[133] Yang Z.-W., Tang C.-E., Zhang J.-L., Zhou Q., Zhang Z.-C. (2019). Stability and Antioxidant Activity of Anthocyanins from Purple Sweet Potato (*Ipomoea batatas* L. *Cultivar Eshu* No. 8) Subjected to Simulated in Vitro Gastrointestinal Digestion. Int. J. Food Sci. Technol..

[134] Tu W., Wang H., Li S., Liu Q., Sha H. (2019). The Anti-Inflammatory and Anti-Oxidant Mechanisms of the Keap1/Nrf2/ARE Signaling Pathway in Chronic Diseases. Aging Dis..

[135] Hwang Y.P., Choi J.H., Yun H.J., Han E.H., Kim H.G., Kim J.Y., Park B.H., Khanal T., Choi J.M., Chung Y.C. (2011). Anthocyanins from Purple Sweet Potato Attenuate Dimethylnitrosamine-Induced Liver Injury in Rats by Inducing Nrf2-Mediated Antioxidant Enzymes and Reducing COX-2 and INOS Expression. Food Chem. Toxicol..

[136] Hussain T., Tan B., Yin Y., Blachier F., Tossou M.C.B., Rahu N. (2016). Oxidative Stress and Inflammation: What Polyphenols Can Do for Us?. Oxidative Med. Cell. Longev..

[137] Jin Q., Liu T., Qiao Y., Liu D., Yang L., Mao H., Ma F., Wang Y., Peng L., Zhan Y. (2023). Oxidative Stress and Inflammation in Diabetic Nephropathy: Role of Polyphenols. Front. Immunol..

[138] Kumar S., Pandey A.K., Lu K.P., Sastre J. (2013). Chemistry and Biological Activities of Flavonoids: An Overview. Sci. World J..

[139] Shibata H., Sakamoto Y., Oka M., Kono Y. (1999). Natural Antioxidant, Chlorogenic Acid, Protects against DNA Breakage Caused by Monochloramine. Biosci. Biotechnol. Biochem..

[140] Xu W., Liu L., Hu B., Sun Y., Ye H., Ma D., Zeng X. (2010). TPC in the Leaves of 116 Sweet Potato (*Ipomoea batatas* L.) Varieties and Pushu 53 Leaf Extracts. J. Food Compos. Anal..

[141] Gan L.J., Yang D., Shin J.A., Kim S.J., Hong S.T., Lee J.H., Sung C.K., Lee K.T. (2012). Oxidative Comparison of Emulsion Systems from Fish Oil-Based Structured Lipid versus Physically Blended Lipid with Purple-Fleshed Sweet Potato (*Ipomoea batatas* L.) Extracts. J. Agric. Food Chem..

[142] Trefts E., Gannon M., Wasserman D.H. (2017). The liver. Curr. Biol. Mag..

[143] Devarbhavi H., Asrani S.K., Arab J.P., Nartey Y.A., Pose E., Kamath P.S. (2023). Global Burden of Liver Disease: 2023 Update. J. Hepatol..

[144] Qian H., Chao X., Williams J., Fulte S., Li T., Yang L., Ding W.X. (2021). Autophagy in Liver Diseases: A Review. Mol. Aspects Med..

[145] Li C., Feng Y., Li J., Lian R., Qin L., Wang C. (2023). Extraction, Purification, Structural Characterization, and Hepatoprotective Effect of the Polysaccharide from Purple Sweet Potato. J. Sci. Food Agric..

[146] Wang W., Li J., Wang Z., Gao H., Su L., Xie J., Chen X., Liang H., Wang C., Han Y. (2014). Oral Hepatoprotective Ability Evaluation of Purple Sweet Potato Anthocyanins on Acute and Chronic Chemical Liver Injuries. Cell Biochem. Biophys..

[147] Ding X., Fan S. (2024). Purple Sweet Potato Polysaccharide Ameliorates Concanavalin A-Induced Hepatic Injury by Inhibiting Inflammation and Oxidative Stress. Phytomedicine.

[148] Sun J., Zhou B., Tang C., Gou Y., Chen H., Wang Y., Jin C., Liu J., Niu F., Kan J. (2018). Characterization, Antioxidant Activity and Hepatoprotective Effect of Purple Sweetpotato Polysaccharides. Int. J. Biol. Macromol..

[149] Zhang M., Pan L.J., Jiang S.T., Mo Y.W. (2016). Protective Effects of Anthocyanins from Purple Sweet Potato on Acute Carbon Tetrachloride-Induced Oxidative Hepatotoxicity Fibrosis in Mice. Food Agric. Immunol..

[150] Hwang Y.P., Choi J.H., Han E.H., Kim H.G., Wee J.H., Jung K.O., Jung K.H., Kwon K.I., Jeong T.C., Chung Y.C. (2011). Purple Sweet Potato Anthocyanins Attenuate Hepatic Lipid Accumulation through Activating Adenosine Monophosphate-Activated Protein Kinase in Human HepG2 Cells and Obese Mice. Nutr. Res..

[151] Kang H., Lee S.G. (2021). Protective Effect of Purple Sweet Potato Leaf (*Ipomoea batatas* Linn Convolvulaceae) against Alcohol-Induced Liver Damage in Mice. Trop. J. Pharm. Res..

[152] Cho A.S., Jeon S.M., Kim M.J., Yeo J., Seo K.I., Choi M.S., Lee M.K. (2010). Chlorogenic Acid Exhibits Anti-Obesity Property and Improves Lipid Metabolism in High-Fat Diet-Induced-Obese Mice. Food Chem. Toxicol..

[153] Shi H., Dong L., Jiang J., Zhao J., Zhao G., Dang X., Lu X., Jia M. (2013). Chlorogenic Acid Reduces Liver Inflammation and Fibrosis through Inhibition of Toll-like Receptor 4 Signaling Pathway. Toxicology.

[154] Liu L., Zhang C., Zhai M., Yu T., Pei M., Du P., Li A., Yan J., Li C., Zhang G. (2023). Effect of Chlorogenic Acid on Lipid Metabolism in 3T3-L1 Cells Induced by Oxidative Stress. Food Biosci..

[155] Ngai M., Mariko T., Kishimoto Y., Iizuka M., Saita E., Toyozaki M., Kamiya T., Ikeguchi M., Kondo K. (2011). Sweet Potato (*Ipomoea batatas* L.) Leaves Suppressed Oxidation of Low Density Lipoprotein (LDL) in Vitro and in Human Subjects. J. Clin. Biochem. Nutr..

[156] Zhao J.G., Yan Q.Q., Xue R.Y., Zhang J., Zhang Y.Q. (2014). Isolation and Identification of Colourless Caffeoyl Compounds in Purple Sweet Potato by HPLC-DAD-ESI/MS and Their Antioxidant Activities. Food Chem..

[157] Tang Y., Cai W., Xu B. (2015). Profiles of Phenolics, Carotenoids and Antioxidative Capacities of Thermal Processed White, Yellow, Orange and Purple Sweet Potatoes Grown in Guilin, China. Food Sci. Hum. Wellness.

[158] Lekmine S., Boussekine S., Akkal S., Martín-García A.I., Boumegoura A., Kadi K., Djeghim H., Mekersi N., Bendjedid S., Bensouici C. (2021). Investigation of Photoprotective, Anti-Inflammatory, Antioxidant Capacities and LC–ESI–MS Phenolic Profile of Astragalus gombiformis Pomel. Foods.

[159] Kim D., Kim Y., Kim Y. (2023). Effect of Purple Sweet Potato Using Different Cooking Methods on Cytoprotection against Ethanol-Induced Oxidative Damage through Nrf2 Activation in HepG2 Cells. Antioxidants.

[160] Chao P.Y., Huang Y.P., Hsieh W. (2013). Bin Inhibitive Effect of Purple Sweet Potato Leaf Extract and Its Components on Cell Adhesion and Inflammatory Response in Human Aortic Endothelial Cells. Cell Adhes. Migr..

[161] Sugata M., Lin C.Y., Shih Y.C. (2015). Anti-Inflammatory and Anticancer Activities of Taiwanese Purple-Fleshed Sweet Potatoes (*Ipomoea batatas* L. Lam) Extracts. Biomed Res. Int..

[162] Jiang T., Zhou J., Liu W., Tao W., He J., Jin W., Guo H., Yang N., Li Y. (2020). The Anti-Inflammatory Potential of Protein-Bound Anthocyanin Compounds from Purple Sweet Potato in LPS-Induced RAW264.7 Macrophages. Food Res. Int..

[163] Sun J., Chen H., Kan J., Gou Y., Liu J., Zhang X., Wu X., Tang S., Sun R., Qian C. (2020). Anti-Inflammatory Properties and Gut Microbiota Modulation of an Alkali-Soluble Polysaccharide from Purple Sweet Potato in DSS-Induced Colitis Mice. Int. J. Biol. Macromol..

[164] Sun R., Kan J., Cai H., Hong J., Jin C., Zhang M. (2022). In Vitro and in Vivo Ameliorative Effects of Polyphenols from Purple Potato Leaves on Renal Injury and Associated Inflammation Induced by Hyperuricemia. J. Food Biochem..

[165] Subawa I.W., Astawa P., Bakta I.M., Astawa I.N.M., Krisna G.A. (2023). Purple Sweet Potato (*Ipomoea batatas* L.) Extract Effects on Levels of Inflammatory Markers and Chondrocyte Count in Gout Arthritis Wistar Rat Model. Foot Ankle Surg..

[166] Lee S.L., Chin T.Y., Tu S.C., Wang Y.J., Hsu Y.T., Kao M.C., Wu Y.C. (2015). Purple Sweet Potato Leaf Extract Induces Apoptosis and Reduces Inflammatory Adipokine Expression in 3T3-L1 Differentiated Adipocytes. Evid.-Based Complement. Altern. Med..

[167] Solihah I., Herlina H., Munirah E., Haryanti H., Amalia M., Rasyid R.S.P., Suciati T., Fatma F. (2023). The Hypoglycemic Effect of Purple Sweet Potato Leaf Fractions in Diabetic Rats. J. Adv. Pharm. Educ. Res..

[168] Martínez-Zamora L., Penalver R., Ros G., Nieto G. (2021). Olive tree derivatives and Hydroxytyrosol: Their potential effects on human health and its use as functional ingredient in meat. Foods.

[169] Shen L., Yang Y., Zhang J., Feng L., Zhou Q. (2005). Diacylated Anthocyanins from Purple Sweet Potato (*Ipomoea batatas* L.) Attenuate Hyperglycemia and Hyperuricemia in Mice Induced by a High-Fructose/High-Fat Diet. J. Zhejiang Univ.-Sci. B.

[170] Mi W., Hu Z., Zhao S., Wang W., Lian W., Lu P., Shi T. (2024). Purple Sweet Potato Anthocyanins Normalize the Blood Glucose Concentration and Restore the Gut Microbiota in Mice with Type 2 Diabetes Mellitus. Heliyon.

[171] Lee C.-L., Lee S.-L., Chen C.-J., Chen H.-C., Kao M.-C., Liu C.-H., Chen J.-Y., Lai Y.-T., Wu Y.-C. (2016). Characterization of Secondary Metabolites from Purple *Ipomoea batatas* Leaves and Their Effects on Glucose Uptake. Molecules.

[172] Wira Mahadita G., Suastika K. (2016). Purple Sweet Potato Tuber Extract Lowers Mallondialdehyde and Improves Glycemic Control in Subjects with Type 2 Diabetes Mellitus. Glob. Adv. Res. J. Med. Med. Sci..

[173] Li J., Shi Z., Mi Y. (2018). Purple Sweet Potato Color Attenuates High Fat-Induced Neuroinflammation in Mouse Brain by Inhibiting Mapk and NF-ΚB Activation. Mol. Med. Rep..

[174] Widyastuti K., Mahadewa T.G.B., Suprapta D.N., Sudewi A.A.R. (2022). Effect of Providing Purple Sweet Potato Water Extract on Tumor Necrosis Factor-α Levels, Protein 53 Expression, Glial Fibrillary Acidic Protein Expression, Brain-Derived Neurotrophic Factor Levels, and Spatial Working Memory in Rats with d-Galactose Induction. Dement. Neuropsychol..

[175] Kang H., Kwak Y.G., Koppula S. (2014). Protective Effect of Purple Sweet Potato (*Ipomoea batatas* Linn, Convolvulaceae) on Neuroinflammatory Responses in Lipopolysaccharide-Stimulated Microglial Cells. Trop. J. Pharm. Res..

[176] Lu J., Wu D.M., Zheng Y.L., Hu B., Zhang Z.F. (2010). Purple Sweet Potato Color Alleviates D-Galactose-Induced Brain Aging in Old Mice by Promoting Survival of Neurons via PI3K Pathway and Inhibiting Cytochrome C-Mediated Apoptosis. Brain Pathol..

[177] Made Oka Adnyana I., Raka Sudewi A., Purwa Samatra D., Suprapta D. (2018). Neuroprotective Effects of Purple Sweet Potato Balinese Cultivar in Wistar Rats With Ischemic Stroke. Open Access Maced. J. Med. Sci..

[178] Shan Q., Lu J., Zheng Y., Li J., Zhou Z., Hu B., Zhang Z., Fan S., Mao Z., Wang Y.-J. (2009). Purple Sweet Potato Color Ameliorates Cognition Deficits and Attenuates Oxidative Damage and Inflammation in Aging Mouse Brain Induced by D-Galactose. J. Biomed Biotechnol..

[179] Sun H., Zhang P., Zhu Y., Lou Q., He S. (2018). Antioxidant and Prebiotic Activity of Five Peonidin-Based Anthocyanins Extracted from Purple Sweet Potato (*Ipomoea batatas* (L.) Lam.). Sci. Rep..

[180] Tang C., Sun J., Zhou B., Jin C., Liu J., Kan J., Qian C., Zhang N. (2018). Effects of Polysaccharides from Purple Sweet Potatoes on Immune Response and Gut Microbiota Composition in Normal and Cyclophosphamide Treated Mice. Food Funct..

[181] Zhang X., Yang Y., Wu Z., Weng P. (2016). The Modulatory Effect of Anthocyanins from Purple Sweet Potato on Human Intestinal Microbiota in Vitro. J. Agric. Food Chem..

[182] Kilua A., Han K.H., Fukushima M. (2020). Effect of Polyphenols Isolated from Purple Sweet Potato (*Ipomoea batatas* Cv. *Ayamurasaki*) on the Microbiota and the Biomarker of Colonic Fermentation in Rats Fed with Cellulose or Inulin. Food Funct..

[183] Kilua A., Nomata R., Nagata R., Fukuma N., Shimada K., Han K.-H., Fukushima M. (2019). Purple Sweet Potato Polyphenols Differentially Influence the Microbial Composition Depending on the Fermentability of Dietary Fiber in a Mixed Culture of Swine Fecal Bacteria. Nutrients.

[184] Zhang Z.C., Zhou Q., Yang Y., Wang Y., Zhang J.L. (2019). Highly Acylated Anthocyanins from Purple Sweet Potato (*Ipomoea batatas* L.) Alleviate Hyperuricemia and Kidney Inflammation in Hyperuricemic Mice: Possible Attenuation Effects on Allopurinol. J. Agric. Food Chem..

[185] Hwa K.S., Chung D.-M., Chung Y.C., Chun H.K. (2011). Hypouricemic Effects of Anthocyanin Extracts of Purple Sweet Potato on Potassium Oxonate-Induced Hyperuricemia in Mice. Phytother. Res..

[186] Zhang Z.C., Wang H.B., Zhou Q., Hu B., Wen J.H., Zhang J.L. (2017). Screening of Effective Xanthine Oxidase Inhibitors in Dietary Anthocyanins from Purple Sweet Potato (*Ipomoea batatas* L. *Cultivar Eshu* No.8) and Deciphering of the Underlying Mechanisms in Vitro. J. Funct. Foods.

[187] Zhang Z.C., Su G.H., Luo C.L., Pang Y.L., Wang L., Li X., Wen J.H., Zhang J.L. (2015). Effects of Anthocyanins from Purple Sweet Potato (*Ipomoea batatas* L. *Cultivar Eshu* No. 8) on the Serum Uric Acid Level and Xanthine Oxidase Activity in Hyperuricemic Mice. Food Funct..

[188] Guo L., Liu J., Yang Y., Zeng Y., Yuan F., Zhong F., Jin Y., Wan R., Liu W. (2021). Purple Sweet Potato Anthocyanins Elicit Calcium Overload-Induced Cell Death by Inhibiting the Calcium-Binding Protein S100A4 in Acute Lymphoblastic Leukemia. Food Biosci..

[189] Vishnu V.R., Renjith R.S., Mukherjee A., Anil S.R., Sreekumar J., Jyothi A.N. (2019). Comparative Study on the Chemical Structure and in Vitro Antiproliferative Activity of Anthocyanins in Purple Root Tubers and Leaves of Sweet Potato (*Ipomoea batatas*). J. Agric. Food Chem..

[190] Ji C., Zhang Z., Zhang B., Chen J., Liu R., Song D., Li W., Lin N., Zou X., Wang J. (2021). Purification, Characterization, and in Vitro Antitumor Activity of a Novel Glucan from the Purple Sweet Potato *Ipomoea batatas* (L.) Lam. Carbohydr. Polym..

[191] Meng M., Sun Y., Qi Y., Xu J., Sun J., Bai Y., Han L., Han R., Hou L., Sun H. (2023). Structural Characterization and Induction of Tumor Cell Apoptosis of Polysaccharide from Purple Sweet Potato (*Ipomoea batatas* (L.) Lam). Int. J. Biol. Macromol..

[192] Martínez L., Ros G., Nieto G. (2020). Effect of natural extracts obtained from food industry by-products on nutritional quality and shelf life of chicken nuggets enriched with organic Zn and Se pro-vided in broiler diet. Poult. Sci..

[193] Asadi K., Ferguson L.R., Philpott M., Karunasinghe N. (2017). Cancer-Preventive Properties of an Anthocyanin-Enriched Sweet Potato in the APC MIN Mouse Model. J. Cancer Prev..

[194] Ryu H.W., Lee S.U., Lee S., Song H.H., Son T.H., Kim Y.U., Yuk H.J., Ro H., Lee C.K., Hong S.T. (2017). 3-Methoxy-Catalposide Inhibits Inflammatory Effects in Lipopolysaccharide-Stimulated RAW264.7 Macrophages. Cytokine.

[195] Kaser A., Zeissig S., Blumberg R.S. (2010). Genes and Environment: How Will Our Concepts on the Pathophysiology of IBD Develop in the Future?. Dig. Dis..

[196] Martínez J., Nieto G., Castillo J., Ros G. (2014). Influence of in vitro gastrointestinal digestion and/or grape seed extract addition on antioxidant capacity of meat emulsions. LWT Food Sci. Technol..

[197] Díaz P., Nieto G., Bañón S., Garrido M.D. (2009). Determination of shelf life of sousvide salmon (*Salmo salar*) based on sensory attributes. J. Food Sci..

[198] De Aguiar Cipriano P., Kim H., Fang C., Paula Venancio V., Mertens-Talcott S.U., Talcott S.T. (2022). In Vitro Digestion, Absorption and Biological Activities of Acylated Anthocyanins from Purple Sweet Potatoes (*Ipomoea batatas*). Food Chem..

[199] Wang Y.J., Zheng Y.L., Lu J., Chen G.Q., Wang X.H., Feng J., Ruan J., Sun X., Li C.X., Sun Q.J. (2010). Purple Sweet Potato Color Suppresses Lipopolysaccharide-Induced Acute Inflammatory Response in Mouse Brain. Neurochem. Int..

[200] Phull A.R., Kim S.J. (2017). Fucoidan as Bio-Functional Molecule: Insights into the Anti-Inflammatory Potential and Associated Molecular Mechanisms. J. Funct. Foods.

[201] Al Bander Z., Nitert M.D., Mousa A., Naderpoor N. (2020). The Gut Microbiota and Inflammation: An Overview. Int. J. Environ. Res. Public Health.

[202] Nie Y., Lin Q., Luo F. (2017). Effects of Non-Starch Polysaccharides on Inflammatory Bowel Disease. Int. J. Mol. Sci..

[203] Hwang S.J., Kim Y.-W., Park Y., Lee H.-J., Kim K.-W. (2014). Anti-Inflammatory Effects of Chlorogenic Acid in Lipopolysaccharide-Stimulated RAW 264.7 Cells. Inflamm. Res..

[204] Astrid Petersmann A., Müller-Wieland D., Müller U.A., Landgraf R., Nauck M., Freckmann G., Heinemann L., Schleicher E. (2019). Definition, Classification and Diagnosis. Exp. Clin. Endocrinol. Diabetes.

[205] Harreiter J., Roden M. (2023). Diabetes Mellitus: Definition, Classification, Diagnosis, Screening and Prevention (Update 2023). Wien. Klin. Wochenschr..

[206] Banday M.Z., Sameer A.S., Nissar S. (2020). Pathophysiology of Diabetes: An Overview. Avicenna J. Med..

[207] European Medicines Agency (2012). Guideline on Clinical Investigation of Medicinal Products in the Treatment or Prevention of Diabetes Mellitus.

[208] Luiz de Brito Alves J., Istri Sri Arisanti C., Made Agus Gelgel Wirasuta I., Musfiroh I., Hainida Khairul Ikram E., Muchtaridi M. (2023). Mechanism of Anti-Diabetic Activity from Sweet Potato (*Ipomoea batatas*): A Systematic Review. Foods.

[209] Nizamutdinova I.T., Jin Y.C., Chung J., Shin S.C., Lee S.J., Seo H.G., Lee J.H., Chang K.C., Kim H.J. (2009). The Anti-Diabetic Effect of Anthocyanins in Streptozotocin-Induced Diabetic Rats through Glucose Transporter 4 Regulation and Prevention of Insulin Resistance and Pancreatic Apoptosis. Mol. Nutr. Food Res..

[210] Kritsilis M., Rizou S.V., Koutsoudaki P.N., Evangelou K., Gorgoulis V.G., Papadopoulos D. (2018). Ageing, Cellular Senescence and Neurodegenerative Disease. Int. J. Mol. Sci..

[211] Agnello L., Ciaccio M. (2022). Neurodegenerative Diseases: From Molecular Basis to Therapy. Int. J. Mol. Sci..

[212] Cheslow L., Snook A.E., Waldman S.A. (2024). Biomarkers for Managing Neurodegenerative Diseases. Biomolecules.

[213] Van Schependom J., D’haeseleer M. (2023). Advances in Neurodegenerative Diseases. J. Clin. Med..

[214] Dugger B.N., Dickson D.W. (2017). Pathology of Neurodegenerative Diseases. Cold Spring Harb. Perspect. Biol..

[215] Ruz C., Alcantud J.L., Montero F.V., Duran R., Bandres-Ciga S. (2020). Proteotoxicity and Neurodegenerative Diseases. Int. J. Mol. Sci..

[216] Kwon H.S., Koh S.-H. (2020). Neuroinflammation in Neurodegenerative Disorders: The Roles of Microglia and Astrocytes. Transl. Neurodegener..

[217] Kowalczyk T., Sitarek P., Zaa C.A., Marcelo Á.J., An Z., Medina-Franco J.L., Velasco-Velázquez M.A. (2023). Anthocyanins: Molecular Aspects on Their Neuroprotective Activity. Biomolecules.

[218] Mattioli R., Francioso A., Mosca L., Silva P. (2020). Anthocyanins: A Comprehensive Review of Their Chemical Properties and Health Effects on Cardiovascular and Neurodegenerative Diseases. Molecules.

[219] Ullah R., Khan M., Shah S.A., Saeed K., Kim M.O. (2019). Natural Antioxidant Anthocyanins—A Hidden Therapeutic Candidate in Metabolic Disorders with Major Focus in Neurodegeneration. Nutrients.

[220] Gibson G.R., Hutkins R., Ellen Sanders M., Prescott S.L., Reimer R.A., Salminen S.J., Scott K., Stanton C., Swanson K.S., Cani P.D. (2017). Expert Consensus Document: The International Scientific Association for Probiotics and Prebiotics (ISAPP) Consensus Statement on the Definition and Scope of Prebiotics. Nat. Rev. Gastroenterol. Hepatol..

[221] Tang C., Han J., Chen D., Zong S., Liu J., Kan J., Qian C., Jin C. (2023). Recent Advances on the Biological Activities of Purple Sweet Potato Anthocyanins. Food Biosci..

[222] Cheng H., Zhang D., Wu J., Liu J., Zhou Y., Tan Y., Feng W., Peng C. (2023). Interactions between Gut Microbiota and Polyphenols: A Mechanistic and Metabolomic Review. Phytomedicine.

[223] Mu J., Xu J., Wang L., Chen C., Chen P. (2021). Anti-Inflammatory Effects of Purple Sweet Potato Anthocyanin Extract in DSS-Induced Colitis: Modulation of Commensal Bacteria and Attenuated Bacterial Intestinal Infection. Food Funct..

[224] Zhang D., Liu J., Cheng H., Wang H., Tan Y., Feng W., Peng C. (2022). Interactions between Polysaccharides and Gut Microbiota: A Metabolomic and Microbial Review. Food Res. Int..

[225] Kwon C., Ediriweera M.K., Kim Cho S. (2023). Interplay between Phytochemicals and the Colonic Microbiota. Nutrients.

[226] Yamauchi T., Ueda T. (2008). Primary Hyperuricemia Due to Decreased Renal Uric Acid Excretion. Nippon. Rinsho. Jpn. J. Clin. Med..

[227] Fathallah-Shaykh S.A., Cramer M.T. (2014). Uric Acid and the Kidney. Pediatr. Nephrol..

[228] Maiuolo J., Oppedisano F., Gratteri S., Muscoli C., Mollace V. (2016). Regulation of Uric Acid Metabolism and Excretion. Int. J. Cardiol..

[229] Yan J., Zhang G., Hu Y., Ma Y. (2013). Effect of Luteolin on Xanthine Oxidase: Inhibition Kinetics and Interaction Mechanism Merging with Docking Simulation. Food Chem..

[230] Lin S., Zhang G., Liao Y., Pan J. (2015). Inhibition of Chrysin on Xanthine Oxidase Activity and Its Inhibition Mechanism. Int. J. Biol. Macromol..

[231] Qian X., Wang X., Luo J., Liu Y., Pang J., Zhang H., Xu Z., Xie J., Jiang X., Ling W. (2019). Hypouricemic and Nephroprotective Roles of Anthocyanins in Hyperuricemic Mice. Food Funct..

[232] Brown J.S., Amend S.R., Austin R.H., Gatenby R.A., Hammarlund E.U., Pienta K.J. (2023). Updating the Definition of Cancer. Mol. Cancer Res..

[233] National Cancer Institute Cancer Statistics. https://www.cancer.gov/about-cancer/understanding/statistics.

[234] Chen P.N., Chu S.C., Chiou H.L., Chiang C.L., Yang S.F., Hsieh Y.S. (2009). Cyanidin 3-Glucoside and Peonidin 3-Glucoside Inhibit Tumor Cell Growth and Induce Apoptosis in Vitro and Suppress Tumor Growth in Vivo. Nutr. Cancer.

[235] Yu X., Zhou C., Yang H., Huang X., Ma H., Qin X., Hu J. (2015). Effect of Ultrasonic Treatment on the Degradation and Inhibition Cancer Cell Lines of Polysaccharides from Porphyra Yezoensis. Carbohydr. Polym..

[236] Jiang C., Xiong Q., Li S., Zhao X., Zeng X. (2015). Structural Characterization, Sulfation and Antitumor Activity of a Polysaccharide Fraction from Cyclina Sinensis. Carbohydr. Polym..

[237] Khoo H.E., Azlan A., Tang S.T., Lim S.M. (2017). Anthocyanidins and Anthocyanins: Colored Pigments as Food, Pharmaceutical Ingredients, and the Potential Health Benefits. Food Nutr. Res..

[238] Tsai T.C., Huang H.P., Chang K.T., Wang C.J., Chang Y.C. (2017). Anthocyanins from Roselle Extract Arrest Cell Cycle G2/M Phase Transition via ATM/Chk Pathway in P53-Deficient Leukemia HL-60 Cells. Environ. Toxicol..

[239] Li W.L., Yu H.Y., Zhang X.J., Ke M., Hong T. (2018). Purple Sweet Potato Anthocyanin Exerts Antitumor Effect in Bladder Cancer. Oncol. Rep..

[240] Tang S., Kan J., Sun R., Cai H., Hong J., Jin C., Zong S. (2021). Anthocyanins from Purple Sweet Potato Alleviate Doxorubicin-Induced Cardiotoxicity in Vitro and in Vivo. J. Food Biochem..

[241] Hanieh H., Gerile C., Narabara K., Gu Z., Abe A., Kondo Y. (2010). In Vivo Immunomodulatory Effects of Dietary Purple Sweet Potato after Immunization in Chicken. Anim. Sci. J..

[242] Miyazaki K., Makino K., Iwadate E., Deguchi Y., Ishikawa F. (2008). Anthocyanins from Purple Sweet Potato *Ipomoea batatas* Cultivar Ayamurasaki Suppress the Development of Atherosclerotic Lesions and Both Enhancements of Oxidative Stress and Soluble Vascular Cell Adhesion Molecule-1 in Apolipoprotein E-Deficient Mice. J. Agric. Food Chem..

[243] Ju R., Zheng S., Luo H., Wang C., Duan L., Sheng Y., Zhao C., Xu W., Huang K. (2017). Purple Sweet Potato Attenuate Weight Gain in High Fat Diet Induced Obese Mice. J. Food Sci..

[244] Kim O.K., Nam D.E., Yoon H.G., Baek S.J., Jun W., Lee J. (2015). Immunomodulatory and Antioxidant Effects of Purple Sweet Potato Extract in LP-BM5 Murine Leukemia Virus-Induced Murine Acquired Immune Deficiency Syndrome. J. Med. Food.

[245] Dong G., Xu N., Wang M., Zhao Y., Jiang F., Bu H., Liu J., Yuan B., Li R. (2021). Anthocyanin Extract from Purple Sweet Potato Exacerbate Mitophagy to Ameliorate Pyroptosis in Klebsiella Pneumoniae Infection. Int. J. Mol. Sci..

[246] Baek S.J., Hammock B.D., Hwang I.K., Li Q., Moustaid-Moussa N., Park Y., Safe S., Suh N., Yi S.S., Zeldin D.C. (2021). Natural Products in the Prevention of Metabolic Diseases: Lessons Learned from the 20th Kast Frontier Scientists Workshop. Nutrients.

[247] Ngcobo A., Mianda S.M., Seke F., Sunette L.M., Sivakumar D. (2024). Phytonutritional Composition and Antioxidant Properties of Southern African, Purple-Fleshed Sweet Potato (*Ipomoea batatas* (L.) Lam.) Storage Roots. Antioxidants.

[248] Xie Y., Liu Q., Mao C., Pang H., Ye P., Cui B., Chen X., Fu H., Wang Y., Wang Y. (2024). Radio Frequency Drying and Puffing of Composite Purple Sweet Potato Chips. J. Food Compos. Anal..

[249] Nurdjanah S., Nurdin S.U., Astuti S., Manik V.E. (2022). Chemical Components, Antioxidant Activity, and Glycemic Response Values of Purple Sweet Potato Products. Int. J. Food Sci..

[250] Rahmi Y., Kurniawati A.D., Widyanto R.M., Ariestiningsih A.D., Aisyi A.Z.A.F., Ruchaina A.N., Sihombing E.V., Istira F.B., Nafsiyah I., Permatasari K.D. (2021). The Sensory, Physical and Nutritional Quality Profiles of Purple Sweet Potato and Soy-Based Snack Bars for Pregnant Women. J. Public Health Res..

[251] Wijaya H., Slay A., Abdullah N. (2021). Ice Cream Products Made from Processed Purple Sweet Potatoes: A Product Organoleptic Study. IOP Conf. Ser. Earth Environ. Sci..

[252] Basílio L.S.P., Vanz Borges C., Minatel I.O., Vargas P.F., Tecchio M.A., Vianello F., Lima G.P.P. (2022). New Beverage Based on Grapes and Purple-Fleshed Sweet Potatoes: Use of Non-Standard Tubers. Food Biosci..

[253] Im Y.R., Kim I., Lee J. (2021). Phenolic Composition and Antioxidant Activity of Purple Sweet Potato (*Ipomoea batatas* (l.) Lam.): Varietal Comparisons and Physical Distribution. Antioxidants.

[254] Amoah I., Cobbinah J.C., Yeboah J.A., Essiam F.A., Lim J.J., Tandoh M.A., Rush E. (2023). Edible Insect Powder for Enrichment of Bakery Products—A Review of Nutritional, Physical Characteristics and Acceptability of Bakery Products to Consumers. Future Foods.

[255] Olson R., Gavin-Smith B., Ferraboschi C., Kraemer K. (2021). Food Fortification: The Advantages, Disadvantages and Lessons from Sight and Life Programs. Nutrients.

[256] Lin S. (2022). Dietary Fiber in Bakery Products: Source, Processing, and Function. Adv. Food Nutr. Res..

[257] Bhatnagar P., Gururani P., Parveen A., Gautam P., Chandra Joshi N., Tomar M.S., Nanda M., Vlaskin M.S., Kumar V. (2024). Algae: A Promising and Sustainable Protein-Rich Food Ingredient for Bakery and Dairy Products. Food Chem..

[258] Kourkouta L., Koukourikos K., Iliadis C., Ouzounakis P., Monios A., Tsaloglidou A. (2017). Bread and Health. J. Pharm. Pharmacol..

[259] Rezende L.V., Paraizo W.B., de Santos J.D.A., Amorim K.A., Goulart G.A.S., Becker F.S., Damiani C. (2024). Study of the Drying Process of the Purple-Fleshed Sweet Potato (*Ipomoea batatas* (L.) Lam) in Spouted Bed. Food Sci. Technol..

[260] Cui R., Zhu F. (2022). Changes in Structure and Phenolic Profiles during Processing of Steamed Bread Enriched with Purple Sweetpotato Flour. Food Chem..

[261] Santiago D.M., Matsushita K., Tsuboi K., Yamada D., Murayama D., Kawakami S., Shimada K., Koaze H., Yamauchi H. (2015). Texture and Structure of Bread Supplemented with Purple Sweet Potato Powder and Treated with Enzymes. Food Sci. Technol. Res..

[262] Kweman N.T., Julianti E., Romauli N.D.M. (2021). Physicochemical Characteristics and Glycemic Index of Bread Made from Purple Sweet Potato Flour, Starch, Fiber from Solid Waste of Starch Processing. IOP Conf. Ser. Earth Environ. Sci..

[263] Zhu F., Sun J. (2019). Physicochemical and Sensory Properties of Steamed Bread Fortified with Purple Sweet Potato Flour. Food Biosci..

[264] Laila U., Rochmadi, Pudjiraharti S. (2019). Microencapsulation of Purple-Fleshed Sweet Potato Anthocyanins with Chitosan-Sodium Tripolyphosphate by Using Emulsification-Crosslinking Technique. J. Math. Fundam. Sci..

[265] Herminiati A., Kurniasari R.A., Cahyadi W. (2023). Optimization of Formula Cookies Based on Purple Sweet Potato Flour (*Ipomoea batatas* L.) Using I-Optimal Mixture Design Method. BIO Web Conf..

[266] Liu Y.-N., Jeong D.-H., Jung J.-H., Kim H.-S. (2013). Quality Characteristics and Antioxidant Activities of Cookies Added with Purple Sweet Potato Powder. Korean J. Food Cook. Sci..

[267] Chung H.J. (2009). Influence of Purple Sweet Potato Powder Addition on the Quality Characteristics and Oxidative Stability of Cookies. J. Food Sci. Nutr..

[268] Muhammad R., Ikram E.H.K., Sharif M.S.M., Nor N.M. (2022). The Physicochemical Analysis and Anthocyanin Level of Malaysian Purple Sweet Potato Cracker. Curr. Res. Nutr. Food Sci..

[269] Julianti E., Lubis Z., Limanto S. (2019). Utilization of Purple Sweet Potato Flour, Starch, and Fibre in biscuits Making. Int. Conf. Food Bio-Ind..

[270] Manalu M., Rumida, Julianti E., Romauli N.D.M. (2024). Composites Flour Formulation Made from Yellow Pumpkin, Purple Sweet Potato, Corn, and Wolf-Herring Flour for Replacement of Wheat Flour on Low- and High- Moisture Foods Part I: Cookies and Muffin. Food Humanit..

[271] Bakar M.F.A., Ranneh Y., Kamil N.F.M. (2022). Development of High Fiber Rich Antioxidant Biscuits from Purple and Orange Sweet Potato Peels. Food Res..

[272] Shewry P.R., Halford N.G., Belton P.S., Tatham A.S. (2002). The Structure and Properties of Gluten: An Elastic Protein from Wheat Grain. Philos. Trans. R. Soc. B Biol. Sci..

[273] Huang Y., Zhou S., Zhao G., Ye F. (2021). Destabilisation and Stabilisation of Anthocyanins in Purple-Fleshed Sweet Potatoes: A Review. Trends Food Sci. Technol..

[274] Nieto G. (2013). Incorporation of by-products of rosemary and thyme in the diet of ewes: Effect on the fatty acid profile of lamb. Eur. Food Res. Technol..

[275] Nieto G., Bañón S., Garrido M.D. (2012). Incorporation of thyme leaves in the diet of pregnant and lactating ewes: Effect on the fatty acid profile of lamb. Small Rumin. Res..

[276] Aziz A.A., Padzil A.M., Muhamad I.I. (2018). Effect of Incorporating Purple-Fleshed Sweet Potato in Biscuit on Antioxidant Content, Antioxidant Capacity and Colour Characteristics. Malays. J. Anal. Sci..

